# Vogt-Koyanagi-Harada disease: review of a rare autoimmune disease targeting antigens of melanocytes

**DOI:** 10.1186/s13023-016-0412-4

**Published:** 2016-03-24

**Authors:** Marcelo Mendes Lavezzo, Viviane Mayumi Sakata, Celso Morita, Ever Ernesto Caso Rodriguez, Smairah Frutuoso Abdallah, Felipe T. G. da Silva, Carlos Eduardo Hirata, Joyce Hisae Yamamoto

**Affiliations:** Uveitis Service, Department of Ophthalmology, Hospital das Clínicas, Faculdade de Medicina da Universidade de São Paulo, Rua Diana, 863 apto 91J, 05019-000 São Paulo, Brazil

**Keywords:** Vogt-Koyanagi-Harada disease, Uveitis, Review

## Abstract

Vogt-Koyanagi-Harada disease (VKHD) is a rare granulomatous inflammatory disease that affects pigmented structures, such as eye, inner ear, meninges, skin and hair. This disease is mainly a Th1 lymphocyte mediated aggression to melanocytes after a viral trigger in the presence of HLA-DRB1*0405 allele. The absence of ocular trauma or previous intraocular surgery sets VKHD appart from sympathetic ophthalmia, its main differential diagnosis. The disease has an acute onset of bilateral blurred vision with hyperemia preceded by flu-like symptoms. The acute uveitic stage is characterized by a diffuse choroiditis with serous retinal detachment and optic disc hyperemia and edema. Fluorescein angiography in this phase demonstrates multiple early hyperfluorescent points. After the acute uveitic stage, ocular and integumentary system pigmentary changes may appear. Ocular findings may be accompanied by lymphocytic meningitis, hearing impairment and/or tinnitus in a variable proportion of patients. Prompt diagnosis followed by early, aggressive and long-term treatment with high-dose corticosteroids is most often ensued by good visual outcomes. However, some patients may experience chronic uveal inflammation with functional eye deterioration. The current review discusses the general features of VKHD, including epidemiology, classification into categories, differential diagnosis and current therapeutic approaches.

## Background

Vogt-Koyanagi-Harada disease (VKHD), initially described as an uveomeningoencephalitic syndrome, is a systemic granulomatous autoimmune disease that targets melanocyte-rich tissues, such as the eye, inner ear, meninges, skin and hair [1].

In 1906, Alfred Vogt in Switzerland first described a patient with premature whitening of eyelashes of sudden onset and bilateral subacute iridocyclitis. Twenty years later, Harada (1926) reported a case series with bilateral serous retinal detachment in association with cerebrospinal fluid (CSF) pleocytosis. Shortly thereafter (1929), Koyanagi published a review article associating unequivocally the posterior eye involvement with auditory and integumentary manifestations. In 1932, Babel suggested that these cases represented a single entity, which was then named Vogt-Koyanagi-Harada Disease [2].

It is speculated that the renowned painter Francisco José Goya y Lucientes (1746–1828) may have presented the disease in his midcareer (1792). Its main features were loss of vision and hearing, ringing in the ears, vertigo, weakness on one side of the body, confusion, abdominal pain and malaise. Goya recovered most of his eyesight, but remained permanently deaf [3].

Thus, VKHD is an uncommon multisystem inflammatory disease characterized by panuveitis, often associated with neurologic and cutaneous manifestations, including headache, hearing loss, vitiligo and poliosis.

## Epidemiology

VKHD is an important cause of noninfectious uveitis affecting, more frequently, individuals of pigmented skin, such as Asians, Middle Easterners, Hispanics and Native Americans. It is very infrequent among persons of African descent [4].

The incidence of VKHD varies. Among all cases of uveitis, it was estimated to represent approximately 7 % in Japan [5], 1–4 % in the United States [6] and 3 % in Brazil [7, 8]; thus ranking, along with Behçet’s disease, as the most prevalent causes of noninfectious uveitis in Brazil [7]. In China, VKHD is one of the most common uveitis entities [9]. In the United States, the incidence of VKHD is approximately 1.5 to 6 per 1 million patients, while in Japan it is seen in approximately 800 new patients each year [1, 10].

Most studies have found that women were affected more frequently than men and that most patients were in the second to fifth decades of life at the onset of the disease. However, children [11, 12] and the elderly may also be affected [13, 14]. Women account for 55 to 78 % of VKHD patients in the United States and approximately 38 % in Japan, showing a global variation in gender predilection [1, 10].

## Pathogenesis

**Fig. 1:**
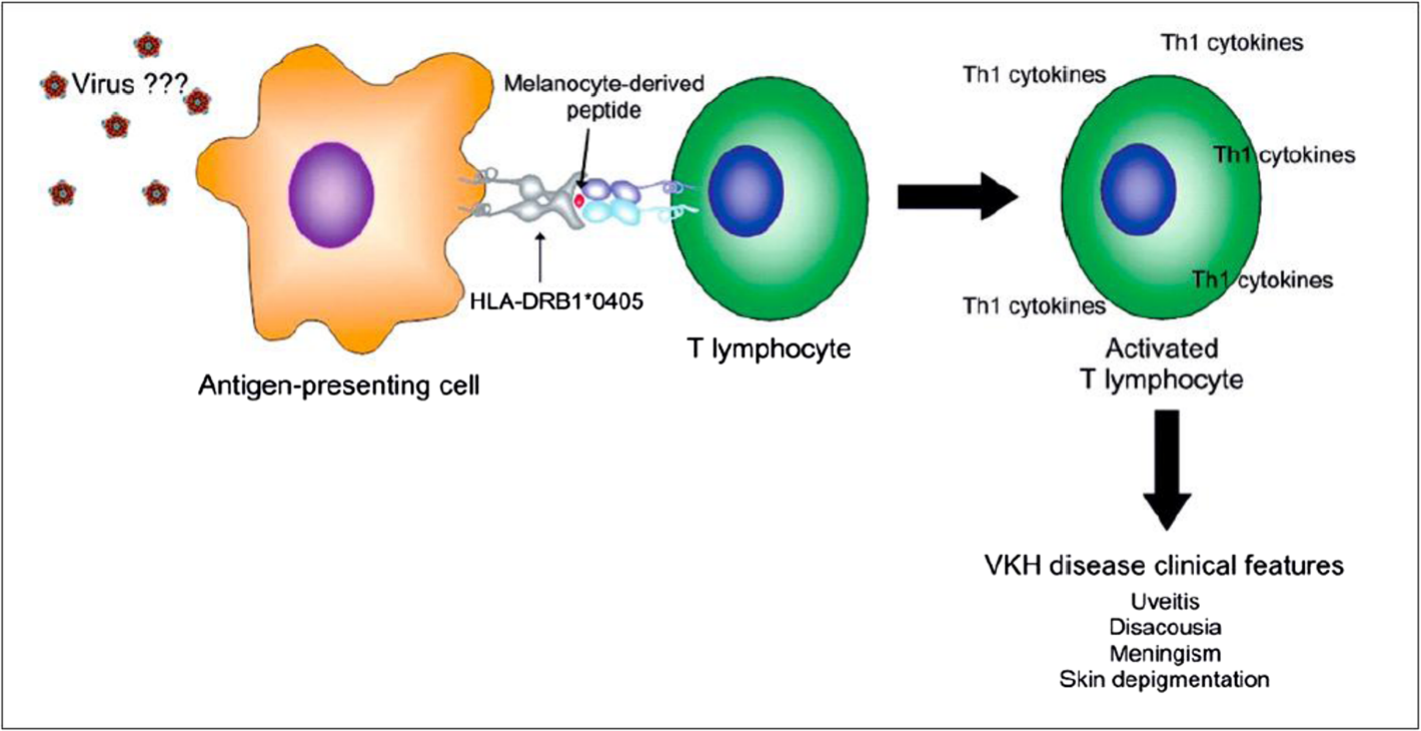
Hypothetical scheme of VKHD pathogenesis. *Courtesy of Arquivos Brasileiros de Oftalmologia* – Damico, F.M., et al., *New insights into Vogt-Koyanagi-Harada disease.* Arq Bras Oftalmol, 2009. 72 [3]: p. 413-20 [39]

The exact etiology of VKHD is still a matter of enquiry. The most accepted mechanism involves an autoimmune aggression against antigens associated with melanocytes in a genetically susceptible individual after a virus infection trigger (Fig. 1). Genome of viruses from the herpes family (Epstein-Barr virus) was detected by PCR (Polymerase Chain Reaction) in the vitreous from VKHD patients [15]. Sugita et al. described that T cells from peripheral blood and intraocular fluid from patients with VKHD cross-reacted with tyrosinase protein and with highly homologous cytomegalovirus specific sequences [16].

Histopathologic findings and in vitro experiments demonstrated the role of CD4^+^ T lymphocytes. Matsuda demonstrated, in eyes globes from patients with VKHD, a close interaction between lymphocytes and melanocytes [17]. In vitro, uveal pigment inhibited leukocyte migration of peripheral blood mononuclear cells (PBMC) from patients with VKHD [18], and both CD4^+^ and CD8^+^ T lymphocytes were cytotoxic against melanocytes in vitro [19]. Moreover, Norose *at al*. described cytotoxicity displayed by lymphocytes from PBMC and CSF of patients with VKHD against the B-36 melanoma cell line [20]. McClellan et al. also found that IL-2 dependent T cells from VKHD patients reacted specifically with normal melanocytes as well as melanoma cells [21].

As suggested by these studies, the immune response is aimed at proteins associated with melanocytes. Melanocyte-specific proteins, shown to have a major role in differentiation, such as tyrosinase (TYR), tyrosinase-related protein 1 (TRP1) and 2 (TRP2), MART-1/Melan A and Pmel17/gp100, are also expressed in human melanoma cell lines and were recognized by T lymphocytes of patients with melanoma and are involved in tumor regression [22]. PBMCs from patients with VKHD recognized peptides derived from tyrosinase family proteins involved in melanin synthesis [23–26]. Peptides derived from TYR, TRP1, and TRP2 induced an autoimmune disease in rats that resembles VKHD [27], making these melanocyte proteins candidate autoantigens for VKHD. Altogether, these data indicate that patients with VKHD are sensitized to melanocyte epitopes and display a peptide-specific Th1 cytokine response [25, 26].

Sakamoto et al., in an immunohistochemical study of eyes affected by VKHD, revealed an increased T helper/T suppressor cells ratio and CD25^+^ and CD26^+^ T lymphocytes within choroidal inflammatory focii. These authors also observed class II major histocompatibility complex (MHC) expression in choroidal melanocytes and endothelium of choriocapillaris [28]. Inomata and Sakamoto demonstrated a remarkable disappearance of choroidal melanocytes in VKHD eyes [29]. These findings suggested that T cell-mediated immune process against melanocytes that express class II MHC play a pathogenic role in VKHD.

Several studies have demonstrated that HLA-DR4 (human leukocyte antigen) is strongly associated with VKHD patients of different ethnic groups, e.g. North Americans [30], Japanese [31–33], Chinese [34], Hispanics [35] and Brazilian [36]. In Japanese subjects, HLA-DRB1*0405 and DRB1*0410 combined susceptibility was robust [relative risk (RR) = 100] [32]. In ethnically heterogeneous Brazilian subjects, HLA-DRB1*0405 was also the predominant allele, with a RR of 12 [36]. Thus, HLA-DRB1*0405 plays a pivotal role in several populations. Besides HLA-DR involvement in VKHD, recently accumulating evidence has demonstrated association of non-HLA genetic factors in VKHD, i.g. cytotoxic T-lymphocyte antigen 4 gene, interleukin genes, macrophage migration inhibitory factor gene and osteopontin gene [37]. More recently, on genome wide analysis, three loci were associated with VKHD susceptibility IL23R-C1orf141, rs117633859; ADO-ZNF365-EGR2, rs442309 and HLA-DRB1/DQA1, rs3021304 [38]. Thus, immunogenetics rather than skin pigmentation may be the clue for disease susceptibility.

Helper T cell subsets producing Th1 cytokines (interferon-gamma and interleukin-2) after melanocyte derived peptides may produce pathological changes in VKHD, such as the granulomatous choroid inflammation in acute phase of VKHD [39–41]. Also of importance, are the cytokines associated with the pro-inflammatory IL-17-producing T helper (Th17) cell, i.g. IL-23, IL-7 and IL-21 [42–44]. In active VKHD patients, increased IL-17 may also result from a decreased IL-27 expression [42, 45]. By contrast, IL-10 and TGF-β regulatory cytokines are associated with the resolution of active inflammation [46]. Active VKHD has also been associated with a diminished function of regulatory T cells (CD4^+^CD25^high^ regulatory) [47]. Concerning innate inflammatory cytokines such as IL-6, Chen et al. described significantly higher levels in aqueous humor of VKHD cataract patients as compared to age-related cataract patients [5, 48].

Cellular and humoral autoimmunity against retinal components have also been demonstrated in patients with VKHD [49–51], as well an anti-Ro/SSA reactivity, in a small percentage of patients [52]. In vitro lymphocytic proliferation in the presence of retinal antigens has shown contradictory results. Naidu et al. showed a positive response to retinal S antigen and interphotoreceptor retinoid binding protein (IRBP) in active untreated patients [49]. Conversely, de Smet et al. detected no such response in chronic VKHD patients [50]. Thus, autoreactivity against retinal proteins seem to differ upon disease stages, i.e., acute vs. chronic. Autoantibodies against photoreceptor outer segments and Müller cells in the sera of VKHD patients have been detected [51]. However, these antibodies could represent a secondary response, which follows the retinal damage in VKHD patients.

## Histopathologic aspects

Histopathological features of VKHD vary according to the stage of disease [53]. The primary pathological feature of VKHD is, however, diffuse thickening of the uveal tract (more prominent in the juxtapapillary choroid). In the acute stage there is a granulomatous process.

In the acute uveitic stage, it is of note a diffuse lymphocytic infiltration with focal aggregates of epithelioid cells and multinucleated giant cells containing pigment devoid apparent choroidal necrosis [53]. Choroidal infiltrate consists of T lymphocytes, which exhibit the markers of helper (CD4^+^) and suppressor/cytotoxic cells, along with melanocytes expressing class II major histocompatibility complex molecules. An eosinophilic exudate with proteinaceous material may be detected underlying the detached retina. While the retinal pigment epithelium (RPE) may appear unharmed using light microscopy, occasional lymphocytes below the RPE may be observed. Focal collections of hyperplastic/modified RPE, macrophages, epithelioid cells and lymphocytes located between RPE and Bruch’s membrane may form the Dalen-Fuchs nodules [53].

During the convalescent stage, there is a nongranulomatous inflammation, which consists hispathologically of a mild to moderate non-granulomatous inflammatory cell infiltrate with focal aggregates of lymphocytes and occasional macrophages. The loss of melanin granules of choroidal melanocytes renders a pale, depigmented aspect to the choroid. Thus, the “sunset glow fundus” appearance in the convalescent stage results from immune-mediated insult to choroidal melanocytes. The RPE may assume either a relatively normal appearance or be focally destroyed, with subsequent chorioretinal adhesions, which correspond to the small atrophic nummular hypopigmented lesions observed in the mid periphery of the fundus [53].

During chronic recurrent stage, a granulomatous choroiditis with damage of choriocapillaris is observed. Furthermore, one may observe a granulomatous infiltrate with less prominent diffuse uveal thickening than that observed in the acute stage. Chorioretinal adhesions with atrophy and/or proliferation of RPE are frequent. Focal areas of hyperpigmentation in depigmented fundi are the consequence of RPE proliferation. This may be accompanied by subretinal neovascularization and elevated pigmented lesions. The hyperplasic RPE can be reorganized and form areas of subretinal fibrosis. Besides these RPE changes, photoreceptor degeneration and gliosis can also be observed. In fact, chronic and recurrent inflammation in the choroid, as noted in VKHD, may stimulate proliferation of retinal pigment epithelial cells [53, 54]. Unlike other stages, there is involvement of the choriocapillaris [53].

In other tissues affected by VKHD (skin and central nervous system (CNS)), there are similar findings: cellular infiltrate composed of T lymphocytes, especially CD4^+^ T cells, and macrophages containing melanin granules [53]. On the other hand, skin lesions of VKHD patients were analysed with electron microscopy and it was possible to demonstrate that, at the periphery of the depigmented lesion, the melanocytes had several subcellular abnormalities, ie, vacuolization of the cytoplasm, aggregation of melanosomes, autophagic vacuoles, fatty degeneration, pyknosis or homogeneous cytoplasmic degeneration, and others. And even absence of melanocytes could be observed [55].

Table 1 describes the main histopathological aspects and their clinical correspondents in Vogt-Koyanagi-Harada disease.

**Table 1:** Histopathological aspects and their clinical correspondents in Vogt-Koyanagi-Harada disease [53, 54]

Histopathologic findings	Clinical correspondents/Ancillary exams
Diffuse granulomatous inflammatory infiltrate of choroid (lymphocytes, macrophages, epithelioid cells, multinucleated giant cells)	In the acute and chronic stages:
	Creamy white lesions in deep retina/choroid
	Diffuse choroid thickening (US, EDI-OCT)
	Dark dots (?) (ICGA)
Dalen-Fuchs nodules (conglomerate between Bruch’s membrane and RPE consisting of lymphocytes, pigment-laden macrophages, epithelioid cells, and proliferated RPE)	In the acute stage:
	Focal leakage at the RPE level (?) (FA)
Choroidal melanocytes without melanin granules	In the convalescent stage:
	“Sunset glow fundus” (diffuse depigmentation)
Focal RPE atrophy with retinal and choroidal adhesion	In the convalescent stage:
	Atrophic nummular hypopigmented lesions in the mid periphery
	Window defect in FA (OCT)
RPE hyperplasia	In the convalescent stage:
	Pigment clumps (OCT, FAF (?))
RPE hyperplasia without melanin granules	Subretinal fibrosis (OCT)
Degenerated photoreceptor	Elipsoidal layer disruption (OCT)
	Abnormal (ERG)

*US* ultrasound, *EDI-OCT* enhanced depth imaging-optical coherence tomography, *ICGA* indocyanine green angiography, *RPE* retinal pigment epithelium, *FA* fluorescein angiography, *FAF* fundus autofluorescence imaging, *ERG* electroretinography

## Diagnostic criteria

The diagnosis of VKHD is primarily based on clinical features. Several criteria have been proposed to clarify the diagnostic approach, including the American Uveitis Society (AUS) in 1978 and the Sugiura’s Criteria in 1976. The AUS adopted the following diagnostic criteria [4, 56]:

- No history of ocular trauma and/or surgery;
- At least three of the following four signs:
  - Bilateral chronic iridocyclitis;
  - Posterior uveitis (multifocal exudative retinal or RPE detachments; disc hyperemia or edema; or “sunset glow fundus”, which is a yellow-orange appearance of the fundus due to depigmentation of the RPE and choroid);
  - Neurologic signs (tinnitus, neck stiffness, cranial nerve or central nervous system symptoms or cerebral spinal fluid pleocytosis);
  - Cutaneous findings (alopecia, poliosis or vitiligo).

The AUS criteria come short in setting appart acute and chronic cases. Another limitation consists in inadequate consideration of acute cases, as two of the four cardinal signs characteristically occur in the convalescent/chronic stages of disease. Moreover, fluorescein (FA) and indocyanine angiography (ICGA), as well ultrasonographic findings were not taken into account by the AUS criteria. As such, neither chronology nor complementary exams were taken into account.

Sugiura et al. proposed another set of diagnostic criteria for VHKD. This system is seldom used outside Japan once CSF analysis is mandatory [4, 57, 58].

More comprehensive criteria were put forth in 2001 by the International Nomenclature Committee, namely the Revised Diagnostic Criteria (RDC). The RDC classifies disease into three categories: complete, incomplete and probable VKH based on the presence of extraocular findings (Table 2) [59]. By considering early and late ocular manifestations, patients may be diagnosed regardless of time elapsed to presentation. However, ancillary examinations (i.e. ICGA [60, 61] and optical coherence tomography (OCT) [62] were not taken into account. Also of note, the RDC does not consider follow up period and treatment; both parameters may interfere on extraocular manifestations incidence [63].

**Table 2:** Revised Diagnostic Criteria of Vogt-Koyanagi-Harada disease proposed by the International Nomenclature Commitee [59]

1. No history of penetrating ocular trauma or surgery preceding the initial onset of uveitis.
2. No clinical or laboratory evidence suggestive of other ocular disease entities.
3. Bilateral ocular involvement (a or b must be met, depending on the stage of disease when the patient is examined):
a. Early manifestations of disease:
I. Evidence of diffuse choroiditis (with or without anterior uveitis, vitreous inflammatory reaction or optic disc hyperemia), which may manifest as (A) focal areas of subretinal fluid, or (B) bullous exudative retinal detachments.
II. If equivocal fundus findings, then both:
A. Fluorescein angiography showing focal delayed choroidal perfusion, multiple areas of pinpoint leakage, large placoid areas of hyperfluorescence, pooling within subretinal fluid, and optic nerve staining;
B. Ultrasonography showing diffuse choroidal thickening without evidence of posterior scleritis.
b. Late manifestations of disease:
I. History suggestive of prior presence of early findings noted in 3a and either (II) or (III) below, or multiple signs from (III) below:
II. Ocular depigmentation: either (A) sunset glow fundus or (B) Sugiura sign.
III. Other ocular signs including (A) nummular chorioretinal depigmented scars, or (B) retinal pigment epithelium clumping and/or migration, or (C) recurrent or chronic anterior uveitis.
4. Neurological/auditory findings (may resolve by time of evaluation):
a. Meningismus (malaise, fever, headache, nausea, abdominal pain, stiffness of the neck and back, or a combination of these factors); note that headache alone is not sufficient to meet definition of meningismus.
b. Tinnitus
c. Cerebrospinal fluid pleocytosis
5. Integumentary findings (not preceding onset of central nervous system or ocular disease):
a. Alopecia, or
b. Poliosis, or
c. Vitiligo.
Complete VKHD: criteria 1–5 must be present
Incomplete VKHD: criteria 1–3 and either 4 or 5 must be present
Probable VKHD (isolated ocular disease): criteria 1–3 must be present

Recently, da Silva et al. demonstrated a correlation between fundus changes and parameters of the full-field electroretinography (ffERG) in patients with VKHD at late stage (with more than 6 months duration of the disease, which includes chronic and convalescent stages). Fundus parameters were used to propose an analytic framework for fundus changes in late-stage VKHD, namely: diffuse pigmentary changes; nummular lesions; pigment clumps and subretinal fibrosis. The correlation of fundus severity and ffERG parameters indicates that fundus changes may reflect functional abnormalities [64].

Lumbar puncture is useful in confirming the diagnosis of VKHD in the acute stage only [44]. Given the large amount of patients found to have auditory symptoms, audiological testing is recommended in VKHD patients [65].

## Clinical features

VKHD is classically divided into four stages: prodromic, acute uveitic, convalescent and chronic/recurrent [1]. To “stage” the disease may enable exchange information rapidly between caregivers as to chronology of the disease which implies in treatment strategies.

### Prodromal stage

The prodromal stage lasts a few days and mimics a viral infection [1]. Patients may present with fever, headache, nausea, vertigo, orbital pain, photophobia, tearing, tinnitus, vertigo and neurologic symptoms. In this stage, cerebrospinal fluid may reveal pleocytosis [4, 59]. Extraocular manifestations will be detailed in an appropriate following section.

### Acute uveitic stage

**Fig. 2:**
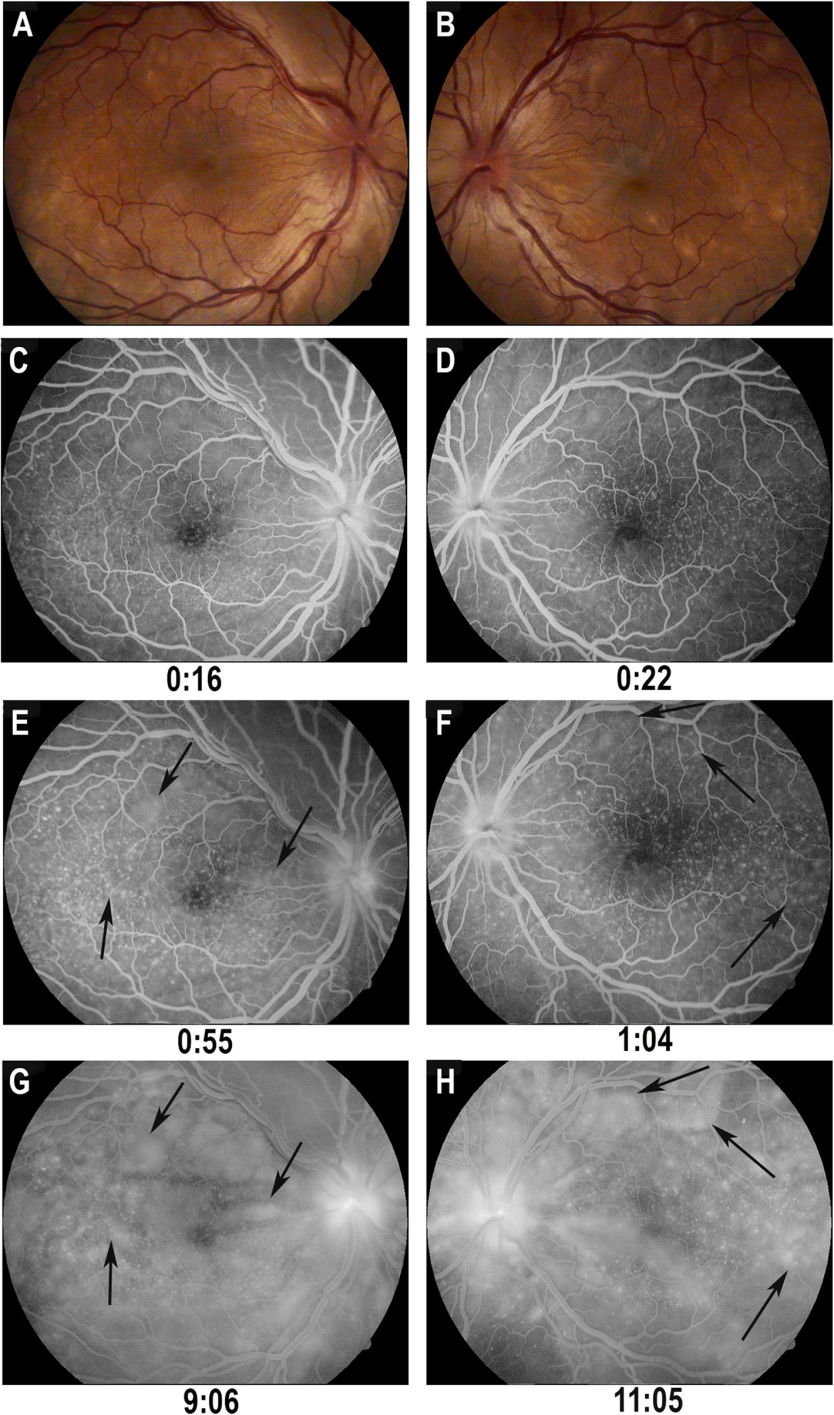
Acute uveitic stage: **a** and **b**: *Right and left* eye of a patient in the acute stage of Vogt-Koyanagi-Harada disease, presenting white- yellowish deep round lesions, hyperemia and blurring of the optic disc and exudative retinal detachment; **c** and **d**: Early fluorescein angiography, showing pinpoints and optic disc hyperfluorescence; **e** and **f**: Increase in pinpoints hyperfluorescence (*arrows*) and optic disc leakage; **g** and **h**: Coalescence of pinpoints hyperfluorescence resulting in pooling of the contrast (*arrows*) in exudative retinal detachment areas

**Fig. 3:**
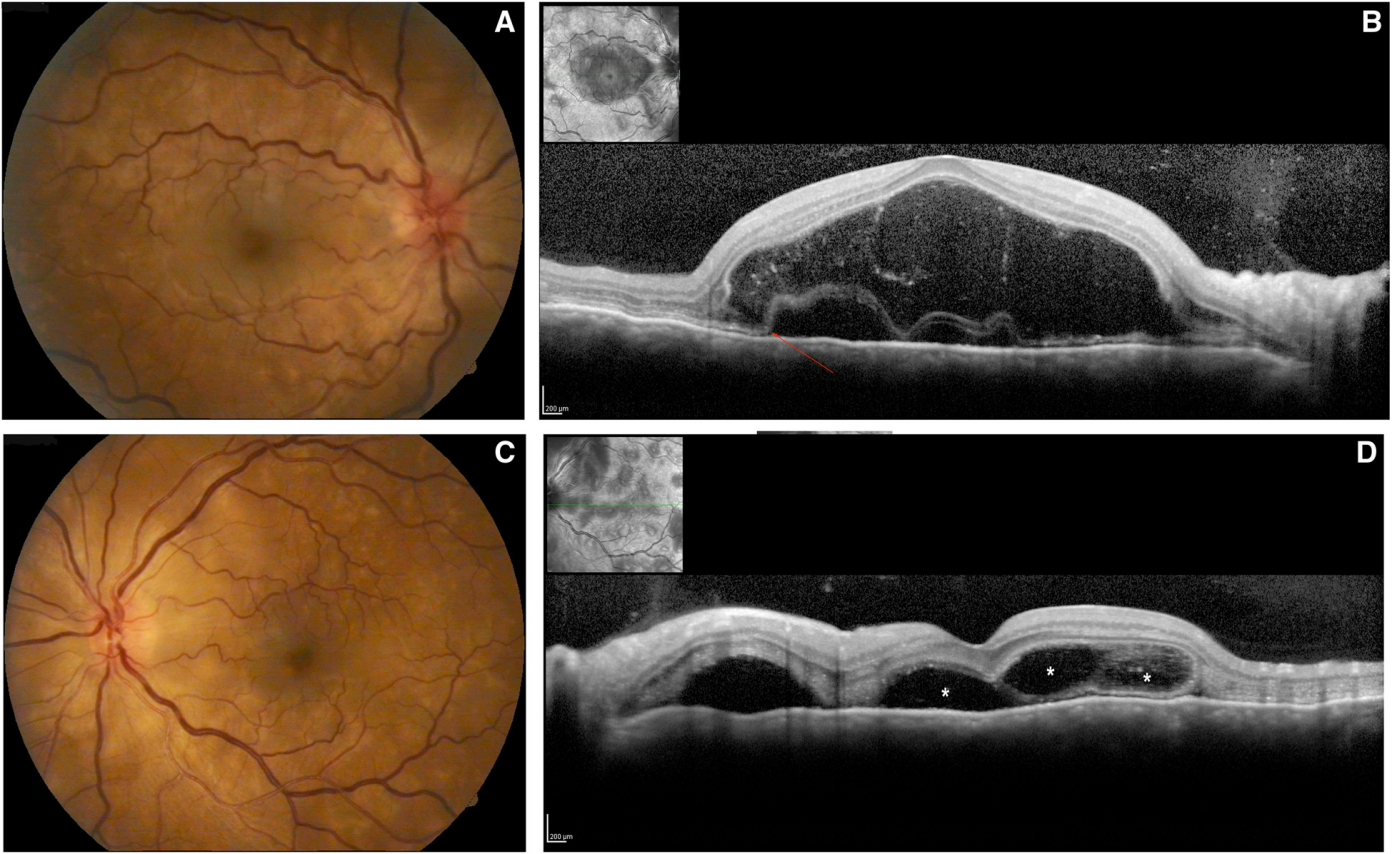
OCT scans in the acute uveitic stage. **a** and **c** Fundoscopic aspect with optic disc swelling and hyperemia, besides multiple yellowish deep round lesions and exudative retinal detachment. **b** OCT scan showing a bullous unique exudative retinal detachment with hyperreflective material within the subretinal fluid (fibrin) and a strand continuous to the ellipsoid zone (*arrow*); **d** OCT scan of an exudative retinal detachment, with multiple compartments (*asterisk*) separated by membranous strutures

This typically occurs within 3 to 5 days of the prodromal stage and lasts for several weeks. Patients may experience acute blurring of vision in both eyes; in 30 % of patients, the involvement of the fellow eye occurs after a few days [1, 4, 58]. The underlying pathologic process in its early stage is the occurrence of a diffuse choroiditis. Features of this choroiditis are exudative detachment of the neurosensory retina secondary to diffuse choroidal inflammation. Hyperemia and edema of the optic disk are observed in about 47 % [4] (Figs. 2 and 3). On FA there are multiple hyperfluorescent leaking dots (**pinpoints**), which become coalescent due to accumulation of fluorescein in the subretinal space (pooling of dye). This is a typical feature of the acute uveitic stage.

Inflammation extends into the anterior segment to varying degrees. Thus, patients with VKHD may have acute bilateral granulomatous iridocyclitis with mutton fat keratic precipitates, iris nodules and shallow anterior chamber due to ciliary body edema and inflammation and suprachoroidal fluid collection. This last feature may lead to acute angle closure glaucoma.

In a Chinese retrospective study including 410 VKHD patients, the posterior and anterior uveal involvement were delineated as consecutive stages [66].

Meningeal involvement and auditory symptoms may also be present during the acute uveitic stage, which may last for weeks or even months.

### Convalescent stage

**Fig. 4:**
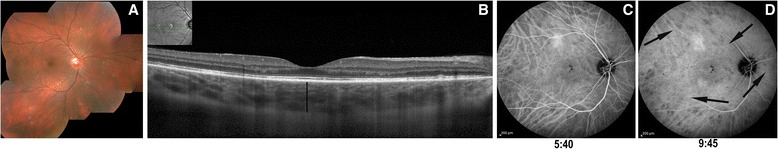
Right eye of a patient in the chronic stage. **a**: Fundoscopy with a mild depigmentation; **b**: OCT scan showing an increased choroidal thickness of 444 μm; **c** and **d**: Indocyanine green angiography showing multiple dark dots (*arrows*) and an uneven background choroidal fluorescence visible at the mid phase of ICGA

The convalescent stage follows the acute uveitic stage, usually a few months later. It is characterized by depigmentation of the integument and choroid. Findings may include vitiligo, alopecia and poliosis. Sugiura described a perilimbal depigmentation that occurs on the first month after the uveitis onset and is observed mainly in Japanese subjects (Sugiura’s sign) [57]. At this stage, varying degree of diffuse or localized depigmentation with areas of pigment accumulation may be observed in the fundus. This depigmentation occurs 2 to 3 months after the uveitic stage; the change may be from brunette to blonde, or it may present as an exaggerated reddish glow fundus [4, 54, 67], which is described as “sunset glow fundus” (Fig. 4). Fundus appearance may have focal accumulation of pigment in bands or in lumps, interspersed with areas of pigment rarefaction. In the mid-periphery, there are multiple, well-defined hypopigmented white lesions.

### Recurrent or chronic stage

This stage may interrupt the convalescent stage. About 17–73 % of patients may progress to recurrence or chronicity [1, 68]. Rubsamen and Gass reported recurrence rates of 43 % within the first 3 months and 52 % within the first 6 months, often associated with rapid tapering of corticosteroids [10]. Recurrence mainly involves anterior segment, without clinically detectable posterior involvement. However, recent studies have shown that there is a persistent aggression to choroidal melanocytes, observed on ICGA and OCT [61, 62, 69, 70].

Ocular complications can often be observed in convalescent and chronic stages. The most frequent ocular complications are cataract, glaucoma, choroidal neovascularization and retinal/choroidal fibrosis.

### Extraocular manifestations

Involvement of integument and the central nervous system (CNS) may be present at various stages of the disease. The frequency and severity of extraocular manifestations varies by patients’ ethnic group, being more common in Asian population (Table 3), and also by treatment adequacy.

**Table 3:** Prevalence of extraocular manifestations in Vogt-Koyanagi-Harada disease

	Japan, 2007 [182]	USA, 1991 [70]	Brazil, 2009 [64]
	*N* = 136 patients	*N* = 48 patients	*N* = 67 patients
Meningeal signs	80 %	67 %	18 %
Pleocytosis	90 %	-	-
Tinnitus	53 %	17 %	24 %
Alopecia	7 %	13 %	9 %
Poliosis	18 %	6 %	22 %
Vitiligo	13 %	10 %	16 %
VKHD categories			
Complete	22 %	-	15 %
Incomplete	65 %	-	55 %
Probable	13 %	-	30 %

#### CNS involvement

The prodromic stage (also called meningeal stage) occurs due to the CNS involvement. During the acute stage meningeal signs may also arise, such as neck stiffness, confusion and headache. CSF pleocytosis is observed in more than 80 % of cases, with a predominance of lymphomononuclear cells, which may be present until the eighth week of the onset of the disease [45]. Serious meningeal-encephalic manifestations and focal neurological signs (i.e.cranial neuropathies, hemiparesis, aphasia, acute transverse myelitis and ciliary ganglionitis) were also reported [4, 58, 71].

#### Inner ear involvement

Changes in the inner ear, such as dysacusis, hearing loss and vertigo, have been observed in 70 % of patients, especially during the prodromal stage. Tinnitus is present in 42 % [65]. The hearing loss pattern is typically cochlear at high frequencies with improvement in 2 to 3 months [63]. Vestibular dysfunction is uncommon.

#### Skin and appendages involvement

**Fig. 5:**
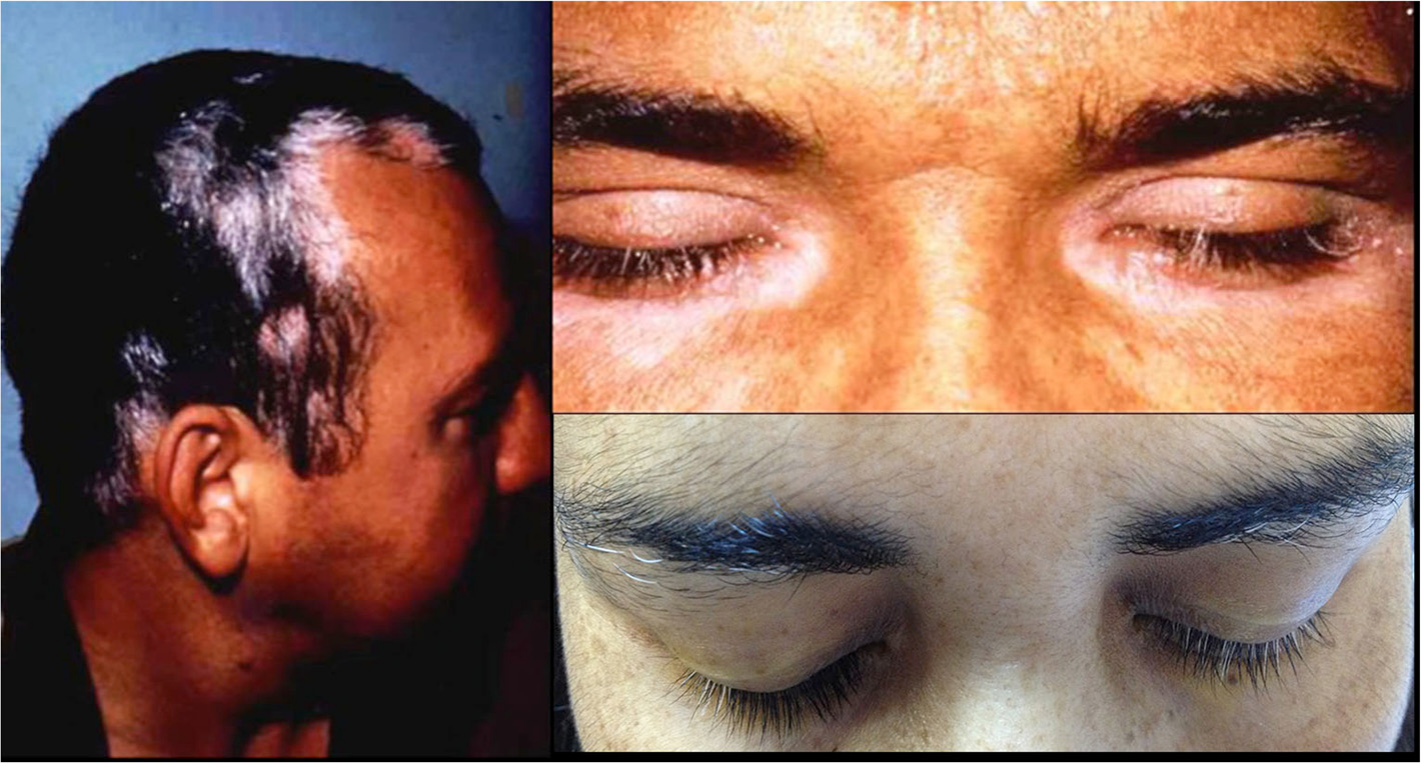
Poliosis of the lashes eyebrows and scalp hair

Cutaneous findings usually develop during the chronic or convalescent stage of the disease and include vitiligo, alopecia and poliosis of the lashes, eyebrows and scalp hair (Fig. 5). Vitiligo can be found in 10 to 63 % of patients [72]. The skin of the back or buttocks seems to be the initial or main anatomic area involved [73].

**Fig. 6:**
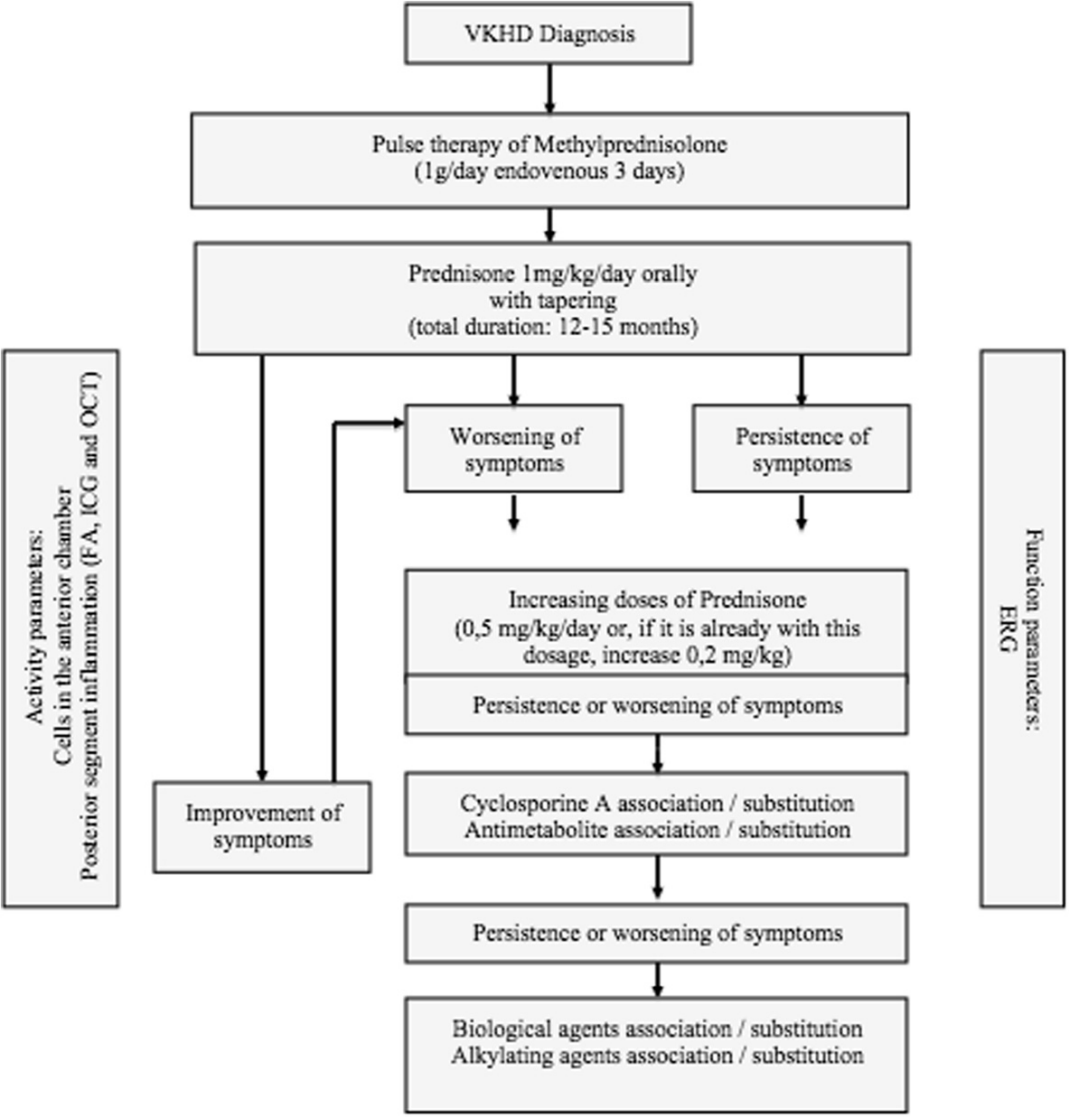
Vogt-Koyanagi-Harada disease treatment flowchart under consideration in the Uveitis Service, Hospital das Clínicas, Faculdade de Medicina, São Paulo University, São Paulo, SP, Brazil

Sugiura’s sign (perilimbal vitiligo) is the earliest depigmentation to occur, presenting itself one month after the uveitic stage [74].

## Differential diagnosis

The main differential diagnosis of VKHD is sympathetic ophthalmia, the latter being necessarily preceded by ocular penetrating trauma and/or previous intraocular surgery. Other conditions that may mimic VKHD are represented in Table 4 [1, 75–82].

**Table 4:** Differential diagnosis of Vogt-Koyanagi-Harada disease

Sympathetic ophthalmia	Previous penetrating ocular trauma or intraocular surgery
Noninfectious choroiditis	Acute posterior multifocal placoid pigment epitheliopathy (APMPPE)
	Birdshot chorioretinopathy
	Multifocal choroiditis and panuveitis (MCP)
	Multiple evanescent white dot syndrome (MEWDS)
Infectious choroiditis	Syphilis
	Tuberculosis
Others inflammatory disorders	Posterior scleritis
	Sarcoidosis
	Lupus choroidopathy
Noninflammatory disorders	Hypertensive choroidopathy
	Bilateral diffuse melanocytic hyperplasia
	Specific hypertensive illness of gestation
	Uveal effusion syndrome
Masquerade syndrome	Carcinoma
	Leukemia
	Lymphoma
	Metastasis

Sympathetic ophthalmia is histopathologically identical to VKHD and can present in a similar fashion with rapid, bilateral visual loss associated with anterior segment inflammation, choroidal thickening, disc hyperemia or edema and serous retinal detachments. Nevertheless, the definition of sympathetic ophthalmia requires previous penetrating trauma or intraocular surgery [1, 83].

Acute posterior multifocal placoid pigment epitheliopathy is a rare inflammatory ocular disease, which affects the choriocapillaris, the RPE and the outer retina in previously healthy and young patients. There is a sudden and painless loss of central visual acuity (VA), unilateral or bilateral, after a viral prodrome, with multiple creamy white lesions, which can evolve to chorioretinal scars. Usually, there is mild or no reaction in the vitreous. FA typically shows early hypofluorescence by blockage in the site of lesions, followed by a late hyperfluorescence. FA findings set VKHD appart from APMMPE. ICGA allows the observation of the entire length of the choroidal involvement as depicted by hypofluorescent lesions in the mid and late phases. Both entities may present with serous retinal detachments, which improve with corticosteroid treatment. Many authors describe CNS involvement that can range from mild changes (as headache) to severe diffuse cerebral vasculitis. Prognosis of APMMPE is generally good with spontaneous and/or rapid resolution of visual function. Some patients may experience only partial visual recovery. Systemic treatment may be indicated in cases with severe visual impairment and/or CNS complications [75, 84–87].

Birdshot chorioretinopathy is a chronic, bilateral intraocular inflammation, occurring more frequently in Caucasians, after the fourth decade of life. The typical presentation is mild yellowish-white lesions throughout the entire posterior pole, cystoid macular edema (CME), disc edema, vasculitis and chronic vitritis. There is mild anterior segment inflammation. It has a chronic course with periods of exacerbation and remission of the disease, with progressive loss of visual acuity. FA findings are not so evident while ICGA tends to show areas of hypofluorescence that persist until the late phases. ERG shows an impairment of rod and cone function. A strong association with HLA-A29 was observed indicating involvement of autoimmune mechanisms in its pathogenesis [78].

Retinal lesions found in multiple evanescent white dot syndrome can be mistaken with VKHD. MEWDS is usually unilateral, affects young women and is characterized by sudden and painless loss of visual acuity. A viral infection may be present in half of the cases. Characteristically, there are numerous, multifocal yellowish-white lesions found in the deep retina/RPE at the posterior pole. In the fovea, there is a distinctive granular appearance. It tends to behave in a self-limited fashion with patients recovering visual acuity in a few weeks. There is usually none or minimal anterior chamber reaction, but vitreous cells may be observed as well choroidal thickening [85]. FA shows hyperfluorescence in the early and late phases, disc leakage and, occasionally, perivascular sheathing. ICGA reveals multiple round hypofluorescent points at the posterior pole. There is enlargement of the blind spot on visual field and reduced amplitude of ERG waves. Recurrences are uncommon [77].

Multifocal choroiditis and panuveitis is a chronic relapsing inflammatory disease, characterized by multiple choroidal lesions on the posterior pole, mid-periphery or periphery, associated with vitritis and anterior chamber reaction. Predominantly, it affects women between 20 and 60 years, in a bilateral and often asymmetrical fashion. FA reveals initial hypofluorescence followed by hyperfluorescence. Subretinal fibrosis and choroidal neovascularization may be observed [88].

Posterior scleritis is an uncommon form of scleral inflammation and is twice as common in women as in men. Thirty percent to 45 % of cases may be associated with systemic diseases such as systemic vasculitis, autoimmune diseases and lymphoma. Posterior scleritis can also present with intense ocular pain, radiating to the head, ears and jaw; redness; choroidal folds, exudative retinal detachment, optic disc edema and choroidal detachment. However, it is usually unilateral and not associated with neurologic or dermatologic findings. Also, unique to posterior scleritis is the ultrasonographic “T sign,” or squaring of the interface between the optic nerve and the sclera, indicating the presence of fluid in the sub-Tenon’s space. Characteristically, there may also be posterior scleral shell thickening and retrobulbar edema. The FA appearance may be similar to the VKHD [1, 79, 89].

Primary intraocular lymphoma is a subtype of non-Hodgkin central nervous system lymphoma (CNSNHL), with moderate to high malignancy, most commonly observed in individuals over 60 years of age. It can affect the vitreous, retina and optic nerve, presenting as a chronic uveitis, little or partially responsive to corticosteroids accompanied by neurological signs and symptoms. Bilateral involvement is common. The fundus may show multifocal elevated subretinal and sub-RPE yellow lesions, involving the posterior pole, associated with vitritis. Satellite lesions may also be present, simulating hypopigmented lesions in the mid-periphery. Generally, there is choroidal thickening with/without associated with retinal detachment. FA shows choroidal fluorescence blockage with late leakage at the site of inflammatory lesions. Unlike VKHD there is extensive focal early hyperfluorescence with dye pooling in the detached retina region in later phases of the angiogram. In patients with CNSNHL, 20 to 25 % have ocular involvement and 56 to 85 % initially presenting ocular lymphoma develop brain lymphoma. Thus, patients with uveitis and neurological symptoms should be carefully examined with CSF tap and neuroimaging (preferably magnetic resonance imaging (MRI) with intravenous Gadollinium enhancement). Diagnosis of intraocular lymphoma can be confirmed by vitreous, retinal and/or choroidal biopsies [76].

Central serous retinopathy (CSC) is an idiopathic condition characterized by the development of serous detachment of the sensory retina and, in some cases, serous detachments of the RPE. It occurs primarily in healthy men between 25 and 55 years of age. In rare cases, these symptoms are accompanied by a migraine-like headache, which could resemble the prodromal stage of VKHD. FA may show different patterns, being the diffuse one more similar to the VKHD findings: large areas of serous detachment and extensive RPE changes. Bilateral CSC may have asymmetric findings. FA show choroidal vascular abnormalities. However, although the yellow-white exudates of VKHD may appear similar to CSC, the granulamatous uveitis is not usually seen in this condition. Besides that, it is very important to differenciate these two patologies, since corticosteroids (the initial and main treatment of VKHD) could increase the risk of developing CSC [90, 91].

Systemic arterial hypertension and pre-ecclampsia may also result in serous retinal detachments. It is suspected that choroidal vascular changes predominate when acute elevation of blood pressure is present, whereas a more gradual onset of hypertension results in retinal vascular changes. Hypertensive choroidopathy may manifest as Elschnig spots (ischemic infarcts of the RPE and hypoperfusion in the underlying choroid); subretinal exudates; serous retinal detachment; fundus depigmentation and optic disc edema [1, 92].

Besides that, the cutaneous manifestations should exclude diagnosis such as Alezzandrini’s syndrome, alopecia areata, vitiligo and piebaldism. Other immunomediated sensorineural hearing loss should also be excluded such as Cogan syndrome. Uncommon VKHD association with cutaneous pigmented malignant melanoma [93], with Chron’s disease [94, 95] and polycystic ovary syndrome [96] among others have been described.

## Ancillary ophthalmic examination

The diagnosis of VKHD is clinical (as no laboratory marker identifies the presence thereof) and is based on the RDC to date (Table 2). Nevertheless, FA and ocular ultrasonography (US) can help the diagnosis and follow-up of atypical cases. ERG examination may be a useful method to evaluate the functional implications of VKHD. Recently, technological advances have allowed a better evaluation of the retina and choroid with ICGA and spectral domain OCT. In conjunction, these imaging modalities have added new parameters for detecting and quantifying inflammation and may allow better assessment of treatment efficacy.

### Fluorescein angiography (FA)

Changes in FA during the acute uveitic stage are characteristic [62, 97] and can help differentiate VKHD from other conditions (Fig. 2). Initially, with active inflammation, FA reveals a delay in choroidal perfusion, causing hypofluorescence of circumscribed areas poorly perfused. The classical numerous hyperfluorescent pinpoints appeared successively and could correspond to focal alterations of the RPE. These hyperfluorescent dots gradually enlarge and stain the surrounding subretinal fluid with pooling of dye in the subretinal space. These focii of hyperfluorescence coincide with areas of choroiditis. Nearly 70 % of the patients may present with disc leakage in the acute stage of disease [97]. In some cases, it is possible to observe areas of linear hypofluorescence, mostly due to the presence of choroidal folds. Sheathing and retinal vascular leakage are rare in contrast to birdshot chorioretinopathy for instance. The presence and extent of pinpoints may be employed in order to monitor the efficacy of initial therapy with corticosteroids [62, 98].

In the convalescent stage, disc leakage and hyperfluorescent dots may also be observed in respectively 29 % and in 14 % of patients [62, 98].

In chronic and recurrent stages, FA may show multiple hyperfluorescent window defects coupled with areas of hypofluorescence due to blockage without progressive staining due to areas of damage to the RPE, rendering a “moth eaten” aspect. Choroidal neovascularization (CNV), retinochoroidal anastomoses and neovascularization of the disc may also be present [62].

### Indocyanine green angiography (ICGA)

ICGA is generally employed in the study of choroidal vasculature and stroma. It may also contribute to the understanding of the pathophysiology of chorioretinal inflammatory disordes [99]. Herbort et al. reviewed the characteristic ICGA signs in VKHD and described the following signs [60–62]:

- Inflammatory choroidal vasculopathy may lead to delay in choroidal perfusion in the early stages of ICGA (2 to 3 min after dye injection), involving the posterior pole and the whole periphery;
- An uneven background choroidal fluorescence visible at the mid phase of ICGA is the result of multiple hypofluorescent round lesions in the choroidal stroma.
- Choroidal folds could appear hyperfluorescent in ICGA.
- Early hyperfluorescence with leakage throughout the choroidal stroma coupled with loss of large choroidal vessels in the intermediate phase (fuzzy vessels) leading to diffuse hyperfluorescence;
- Numerous hypofluorescent, evenly distributed focii (dark dots) in the intermediate phase, that gradually become isofluorescent in the late phase may represent choroidal granulomas (Fig. 3);
- Optic disc hyperfluorescence.

At this point, it is worthy to mention that, till recently, disease activity parameters in non-acute VKHD stage were essentially clinical; nowadays, there is a trend towards taking posterior segment imaging inflammatory signs also into consideration as inflammation markers and, consequently, as indicators of additional systemic treatment.

### Fundus autofluorescence (FAF)

FAF reflects functional and metabolic changes in RPE visualizing lipofuscin (BL-FAF) or melanin or its compounds (NIR-FAF). These two modalities of fundus autofluorescence (FAF) differ according to the wavelength employed i.e. FAF with short wavelength light or blue (BL-FAF) and near-infrared light (NIR-FAF) emission.

Koizumi et al. described that areas of both BL- as well as NIR-FAF hypoautofluorescence in five patients (ten eyes) with acute VKHD corresponded to regions of serous retinal detachment. After resolution of subretinal fluid, FAF demonstrated placoid regions of hyperautofluorescence in the macula and peripapillary region, which corresponded to hypofluorescence on ICGA. In patients receiving initial corticosteroid pulsetherapy, hyperautofluorescent areas resolved, while patients receiving delayed treatment tended to present persistent areas of macular hyperautofluorescence [100] which may be attributed to alterations in distribution of both, melanin as well as lipofucsin.

Heussen et al., in a retrospective study in ten patients (20 eyes) with chronic VKHD utilizing ultra-wide-field FAF, demonstrated peripheral changes on FAF images with no correspondence to color images, i.e. areas of hypoautofluorescence, areas of hyperautofluorescence and “lattice-like” pattern of FAF [101].

Thus, as VKHD may affect both the choroid as well as the RPE, different FAF patterns in both BL- and NIR modalities are not surprising.

### Optical coherence tomography (OCT)

OCT has revealed unique features of multifocal serous retinal detachment in acute VKHD with cystic spaces and membranous structures continuous to the ellipsoid zone (internal and external segments junction of the photoreceptors). Splitting of retinal layers was visible over the IS/OS line near cystoid spaces that involved the fovea in 45 % of the examined eyes. All of these abnormal features were seen below the line representing the external limiting membrane, that is, in the outer photoreceptor layer [102]. Some authors hypothesized that these strands are formed by fibrin and disrupted outer segment of the photorreceptors [102–104] Other OCT findings in the acute stage are intraretinal edema, choroidal folds or striations (RPE undulations), choroidal hyperreflective dots, among others [105–107] (Fig. 3). Prompt resolution of the retinal detachment is usually observed in OCT after high-dose systemic corticosteroid [103, 108]. Several OCT parameters have been associated with a worse prognosis, e.g. “splitting off” of outer segments of the photoreceptor layer from the inner segments [102], height of serous retinal detachment [104], RPE undulations [104].

OCT imaging in chronic stage VKHD showed RPE and outer retinal changes. These coincided with histopathological findings, such as RPE cell clumping and damage to outer- and inner- segment junction of photoreceptors. Vasconcelos-Santos et al. studied patients with chronic VKHD and “sunset glow” fundus. Spectral domain-OCT demonstrated normal retinal architecture in regions of “sunset glow fundus”, RPE/Bruch’s membrane’s thinning in regions of atrophy and RPE/Bruch’s membrane thickening in areas overlying pigmented scars [109].

Enhanced-depth imaging spectral-domain OCT (EDI-OCT) improved ability to visualize the choroid and its thickeness [109, 110]. Patients in the acute uvetic stage had markedly thickened choroids related to inflammatory infiltration and increased exudation [111, 112]. Choroidal thickeness decreases quickly with corticosteroid treatment [111, 112]. Da Silva et al. described that patients with long-standing VKHD presented with thinner choroids when compared to normal individuals [113]. Furthermore, patients with recurrent inflammation presented thicker choroids when compared to patients with quiescent disease [113, 114]. Choroidal thinning at the foveal center occurred in an inversely proportional relation to the duration of disease [113]. EDI-OCT is a non-invasive and quantitative method and can be used to assess the degree of choroidal inflammatory reactions during the follow-up [114].

### Ocular ultrasonography (US)

Echography may be an invaluable adjunct in diagnosis since it allows differentiation with posterior scleritis, benign reactive lymphoid hyperplasia of the uvea, diffuse malignant melanoma and choroidal involvement in leukemia or lymphoma [97, 115].

Ultrasonography may also be helpful when fundus viewing is obscured by media opacities, when presentation is atypical and/or when extraocular signs are absent. High-definition ocular US may demonstrate choroidal thickening in subclinical VKHD and may also aid in monitoring response to treatment, most notably in the presence of media opacities. However, US imaging does not add much information in cases with subtle alterations due to its imaging resolution (100 μm vs 7 μm in SD-OCT and 5 μm in EDI-OCT) Nonetheless, Forster et al. described the following US signs in VKHD [115]:

1. Diffuse choroidal thickening with low to medium reflectivity;
2. Serous retinal detachments around posterior pole or inferiorly;
3. Vitreous opacities without posterior vitreous detachment (PVD);
4. Scleral or episcleral thickening.

Ultrasound biomicroscopy (UBM) allows detailed evaluation of changes affecting the ciliary body and iris. Anterior chamber shallowing was shown to occur acutely due to ciliochoroidal detachment and ciliary body thickening. These may lead to anterior displacement of the iridolenticular diaphragm, simulating acute angle-closure glaucoma [116].

### Electrophysiological testing

Electroretinogram (ERG) can be helpful in monitoring disease course as well as in demonstrating the degree of functional compromise due to the inflammatory damage to retinal components [117, 118]. Abnormal ERGs have been described in VKHD patients with extensive chorioretinal atrophy. Da Silva et al. demonstrated a correlation between fundus changes and parameters of the ffERG in patients with VKHD at late stage (with more than 6 months duration of the disease, which includes chronic and convalescent stages). Patients with more severe fundus-based disease presented greater retinal dysfunction, showing congruity between fundus appearance and the extent of retinal damage. These authors observed diffusely diminished amplitudes in both scotopic and photopic phases, while sparing the respective implicit times [64].

## Management of Vogt-Koyanagi-Harada disease

Treatment of iridocyclitis should be performed according to the intensity of anterior segment inflammation. Topical corticosteroids (e.g., dexamethasone 0.1 % or prednisolone acetate 1 % eyedrops) in combination with mydriatics/cycloplegics (e.g., tropicamide 1.0 % eyedrops) to reduce ciliary spasm and prevent posterior synechiae, are most frequently used [1].

The mainstay of treatment of VKHD is prompt, high-dose systemic corticosteroids, administered either orally (prednisone 1–1.5 mg/kg per day) or through a short course of intravenous delivery (methylprednisolone 1000 mg per day, intravenously, during 3 days), followed by slow tapering of oral corticosteroids throughout a minimum 6-month period (Fig. 6). Timing to initiate therapy, corticosteroid dosing and duration of therapy are the key factors in reducing the chance of recurrences.

Read et al. compared the use of oral corticosteroids and the use of pulsetherapy followed by oral corticosteroids and suggested that the route of initial administration did not influence the outcomes measured by visual acuity [119]. However, more recent studies show a trend to aggressive high-dose corticosteroid, i.e. pulsetherapy, with faster resolution of clinical inflammatory signs as well as posterior segment imaging inflammatory signs (PSIIS). Kawaguchi et al. demonstrated that medium-dose systemic corticosteroid therapy may be insufficient to adequately suppress ocular inflammation in VKHD. In their case series, ICGA detected the persistence of hypofluorescent dark dots after 4 months in patients receiving such regimens. Furthermore, these same patients were more likely to develop “sunset glow fundus”. Dose as high as 0.75 mg/kg per day was necessary during the first 4 months of treatment [120]. Moreover, Chee et al. have emphasized with about equal importance the early treatment instauration [121]. This group demonstrated that peripapillary atrophy (PPA) developed more frequently as well as to a greater extent in patients receiving delayed and low dose corticosteroids [121]. The same authors showed that this finding was a marker of more severe retinal dysfunction in these patients as measured by mfERG [117].

At this point, it is needed to emphasize that a more comprehensive approach of inflammation in post-acute uveitic stage of VKHD does not take into account isolate visual acuity, but also the presence of cells in anterior chamber and PSIIS (FA, ICGA, OCT) [61, 103, 108, 112–114]. Therefore, concerning ICGA signs, hypofluorescent dark dots may resolve in 4 months, while changes in choroidal vascular permeability tend to disappear in 8 weeks due to their response to initial therapy with high-dose corticosteroids [61, 62] Dark dots are the most constant and most readily recordable angiographic sign enabling choroidal inflammatory activity semi-quantitative assessment (Fig. 4).

Some authors indicate therapy guided by ICGA inflammatory signs [68]. Nevertheless, a more comprehensive understanding of choroidal inflammatory signs is lacking [122, 123].

Rapid discontinuation of systemic corticosteroid may incur in recurrences [10, 124]. Several studies have pointed out that the minimum treatment period is 6 months. Lai et al. and Errera et al. formally demonstrated in their retrospective studies the relevance of a minimum of 6-month systemic and/or immunosuppressive drug therapy in diminishing recurrence frequency and severity [124, 125]. The final treatment duration varies widely according to the presence of inflammation.

Immunosuppressive therapy is formally indicated in corticosteroid refractory or intolerant cases [126]. Nevertheless, in VKHD, the long-term systemic corticotherapy was acceptable due to till recent notion that it presents a good response to this therapy alone and had a good prognosis counterbalancing the more serious immunosuppression induced side effects [127]. However, recent literature is pointing out to the deleterious effect on visual function of chronic relentless choroidal inflammation and a trend to an earlier start on systemic immunosuppression.

Some investigators have suggested immunosuppression with agents such antimetabolites, cyclosporine and biological agents (IMT) as first-line therapy in the treatment of VKHD. Aggressive treatment may result in fewer complications and less recurrence. Paredes et al. described IMT given within 6 months of diagnosis with or without steroid was associated with a superior visual outcome when compared to steroid as monotherapy or with delayed additional of IMT [128]. Rao et al. pointed out that a prospective study should be conducted to validate the role of first-line immunosuppressive therapy in all VKHD patients, particularly during the acute stage of the disease, accounting for both, potential side effects as well as little evidence available [122].

Either as first-line IMT or as an adjuvant treatment in chronic recurrent or steroid intolerant cases, use of several IMT drugs has been reported. The choice of immunosuppressive agent will be more dependent on drug availability, including cost, and tolerability than on drug-specific efficacy on VKHD (Table 5). Therefore, much experience has been gained with the wide spread-use of cyclosporine A during the 1980s. However, cyclosporine monotherapy did not demonstrate to be superior to other IMT drugs being modestly effective for controlling ocular inflammation with frequent side effects, mainly with increasing age [129]. Of note is the long list of cyclosporin drug interactions that may increase (e. g., macrolide antibiotics, antifungals, diltiazem, metoclopramide, oral contraceptives, allopurinol etc.) or decrease (e. g., barbiturates, carbamazepine, rifampin etc.) cyclosporine A bioavailability [129].

**Table 5:** Treatment of Vogt-Koyanagi-Harada disease: drugs, dosages and main side effects [126, 127]

Drug	Dosage	Main side effects
Corticosteroids		
Prednisone or prednisolone	1–1.5 mg/kg/day, gradual tapering along a minimum 6 month-period	hyperglicemia, hypertension, osteoporosis, Cushingoid appearance, myopathy, cataract, glaucoma
Pulsetherapy of methylprednisolone	1 g/day during 3 days, followed by oral prednisone	
Antimetabolites		
Azathioprine	1–2.5 mg/kg/day	myelosuppression, gastrointestinal disturbances, infection
Methotrexate	7.5–25 mg/week	nausea, vomiting, abnormalities in liver function tests, infection
Mycophenolate mofetil	1–3 g/day	gastrointestinal disturbances with diarrhea, infection
Calcineurin inhibitors		
Cyclosporine A	up to 5 mg/kg/day, trough 0.1–0.2 μg/L (whole blood)	nephrotoxicity, hepatotoxicity, hyperglycemia, hypertension, hirsutism, gum hyperplasia and neurological disorders
Biological agents		
Infliximab (intravenous)	5 mg/kg at 0,2 and 6 weeks, followed by 5 mg/kg every 6–8 weeks	reactivation of latent tuberculosis, lymphoproliferative diseases
Adalimumab (SubQ)	40 mg every other week	headache, infection
Alkylating agents		
Cyclophosphamide	1–2 mg/kg/day	myelossupression, hair loss, nausea, vomiting, loss of fertility, cancer risk, infection, bladder toxicity

Antimetabolites are drugs that inhibit nucleotide synthesis, inhibiting the division and proliferation of inflammatory cells, i.e. methotrexate, azathioprine and mycophenolate mofetil [130]. Methotrexate has been used to control pediatric VKHD as well as adult VKHD [131]. Mycophenolate mofetil, a selective inhibitor of inosine monophosphate dehydrogenase (an enzyme essential for the proliferation of B and T lymphocytes), has been used as first-line therapy in a prospective study including 19 patients leading to less recurrences and improved visual outcome [132]. Interestingly, Urzua et al. recently evaluted, in a retrospective study, VKHD patients treated with early IMT (within 6 month of diagnosis) and patients treated with late IMT and found no differences in terms of visual acuity improvement, complications and glucocorticoid sparing effect [133]. However, these authors suggested that those with poor response to glucocorticoid therapy could benefit with the IMT as first-line therapy and pointed out that treatment should be individualized. Azathioprine had been demonstrated to be effective by other authors in patients with corticosteroid intolerance or uncontrolled inflammation [134].

Case series demonstrating the efficacy of several other treatment modalities are found in the literature including biologics agents, such as infliximab and rituximab [135–137] and intravitreal drug therapy, such as triamcinolone, bevacizumab and fluocinolone acetonide [138–141]. Even though intravitreal drug therapy as a first line treatment for acute VKHD is very controversial, it may be useful as an adjunt treatment in chronic and/or recurrent stages of the disease.

In infants, methotrexate is most widely used than other IMT and seems to be effective with minimal side effects [11, 142–144]. In pregnancy, high-dose corticosteroids have been used to treat VKHD successfully during the second and third trimester of pregnancy, usually devoid complications in delivery [145–148].

New highly effective drugs with less toxicity in the management of VKHD are continuously being searched. One example is the strong steroid difluprednate, which was used topically, at the onset of diagnosis, and promoted complete resolution of exudative detachments with improvement of visual acuity [149]. New biological agents are also being pursued such as secukinumab and gevokizumab. Secukinumab is a full or recombinant monoclonal antibody against IL-17 and gevokizumab is a humanized IgG2k monoclonal antibody, which binds to IL-1ß with high affinity and inhibits IL-1**β**-mediated responses [150].

## Complications

Chronic or recurrent inflammation may be associated with the development of ocular complications, e.g. cataract, glaucoma and choroidal neovascularization (CNV) (Table 6). Other less common complications have been reported [151–154], including cystoid macular edema, pseudotumoral RPE proliferation, band-shaped keratopathy, optic disc atrophy and phthisis bulbi.

**Table 6:** Prevalence of the most common complications in VKHD

Author	Number of eyes	% Cataract	% Glaucoma	% CNV
Ohno [6]	102	30	20	Not available
Snyder [56]	40	16	Not available	2,5
Rubsamen [10]	52	36	45	9
Forster [183]	84	Not available	38	Not available
Moorthy [1]	130	38	Not available	12
Read [162]	101	42	27	11

Da Silva et al. divided VKHD patients in two groups, i.e. early stage and late stage groups. Patients who presented with symptoms for less than 4 weeks were grouped as early stage; others were grouped as late stage. Patients first seen in the late stage of disease had more ocular complications and relapses after disease onset, when compared to those first seen in the early stage [64].

### Cataract

Cataract may arise due to chronic inflammation and/or prolonged corticosteroid therapy [155]. Cataract formation was reported in 10–42 % of patients. Cataract surgery should be postponed until the uveitis is inactive for a minimum period of 3 months [155–158]. Systemic corticosteroids (0.5–1 mg/kg/day) should be given starting 1 to 2 weeks [159] before surgery and then tapered after surgery accordingly to the intensity of inflammation.. Synechiolysis with or without iris-stretching maneuvers or iris hooks may be required. Foldable hydrophobic acrylic or heparin-surface modified polymethylmethacrylate intraocular lenses (IOL) can be safely used in eyes with VKH disease [156, 158]. Moorthy et al. reported that 68 % of 19 eyes that underwent cataract surgery have a visual acuity of 20/40 or better [155]. In-the-bag intraocular lens implantation has ultimately shifted the paradigm regarding complicated cataract surgery, showing promising results [160, 161].

### Glaucoma

Intraocular pressure (IOP) elevation in patients with VKHD may occur as a consequence of inflammation of the trabecular meshwork, blockage of trabecular meshwork by inflammatory cells, presence of peripheral anterior synechiae and pupillary block with angle closure, among others. Its prevalence ranges widely, from 6 to 45 % [162], which may be attributable to distinct patient populations and follow-up periods. Takahashi et al., in a retrospective study of 217 patients with uveitic glaucoma, found that 16 % of cases were VKHD patients; the majority had active anterior uveitis at the time of high IOP [163]. Medical therapy should be tried, but often these patients end up evolving to trabeculectomy. Iwao et al., in a comparative retrospective study of 101 eyes with uveitic glaucoma and 103 eyes with primary open-angle glaucoma who underwent trabeculectomy with mitomycin C, with an average of 24 months follow-up, reported a success rate of 71 % in patients with uveitis and 90 % in the control group [164].

### Choroidal neovascularization (CNV)

CNV may develop from inflammatory damage to Bruch’s membrane and choriocapillaris, which leads to choroidal and outer retinal ischemia. The ischemia may then serve as a stimulus for proliferation of choriocapillaris endothelium. Moreover, it has been suggested that active inflammation induced the release of chemokines that induces angiogenesis. The prevalence of this complication varies from 7 to 15 % of cases and is associated with poor visual prognosis. They are mainly localized peripapillary and subfoveal where inflammatory foci tend to concentrate [162]. Factors predisposing for the development of CNV development include chronic/recurrent inflammation of the anterior segment and extensive RPE changes. Treatment consists in control of inflammation with corticosteroids and immunosuppressive drugs as well as use of anti-VEGF agents (vascular endothelial growth factor) [165–167]. The need of anti-VEGF reinjection should be evaluated according to the presence of disease activity as estimated on FA, ICGA and/or OCT (presence of intra- or subretinal fluid). Mansour et al., in a retrospective multicenter study, evaluated the visual result of intravitreal injection of bevacizumab in patients with CNV with a 24 months follow-up. They reported aggressive CNV in six cases of VKHD-associated CNV requiring a careful monitoring, systemic immunomodulation and frequent injections of anti-VEGF [167]. Some additional treatment options that warrant investigation in the management of VKHD-associated CNVs include photodynamic therapy, laser photocoagulation and combination of pharmacotherapy of anti-VEGF compounds alongside immunosuppressants [168, 169]. It remains to be determined whether agressive immunossupression or anti-VEGF therapy play the foremost role in the treatment of CNV associated with VKHD.

### Subretinal fibrosis

Subretinal fibrosis is described in 8 to 40 % of VKHD cases and is more common in long standing or recurrent cases. The most common locations are peripapillary and extrafoveal areas. Histopathologic findings reveal the presence of subretinal fibrosis, RPE cells metaplasia and choroidal inflammatory cell aggregates in subretinal fibrosis area. It is believed that cytokines, immunoglobulins and cellular mediators produced by T-lymphocytes cause fibrous tissue production through interaction with the RPE cells, Müller cells and choroidal fibrocytes. Presence of subretinal fibrosis in patients with VKHD is associated with a poor visual prognosis [170, 171]. Kuo et al. observed subretinal fibrosis more often in Hispanics, who develop this complication in a much shorter disease duration than non-Hispanics (median of 6.5 months in Hispanics and 6.5 years for non-Hispanics) [172].

## Prognosis

The visual outcome in patients with VKHD has improved considerably with the use of high-dose corticosteroids, immunosuppressant drugs and advances in the management of complications, such as cataract, glaucoma and CNV. The prognosis of VKHD is usually considered good with 60 % of patients having VA better than 20/40 [1, 10].

However, several evidences point to VKHD as a much more serious illness, i.g. more than 50 % of patients evolves towards chronicity [1, 68, 173] and 50 % of eyes with VKHD develop at least one complication [160]. Furthermore, a more comprehensive approach of inflammation in VKHD does not take into account isolate visual acuity and characterization of inflammatory activity based only on the presence of cells in anterior chamber seems not to be sufficient [69]. Recent choroid imaging advances have made possible a better identification and quantification of inflammatory activity [61, 102, 107, 111–113].

Until recently, VKHD was considered “cured” for those patients who were in the convalescent stage. Nevertheless, recent studies have pointed to disease progression, even in seemingly quiescent cases. The main evidences of this progression are: progressive fundus depigmentation, even in patients without obvious persistent clinical activity [174]; worsening of visual acuity complaints in patients apparently “cured” and with no clinical disease activity [175]; and, presence of inflammatory cells in the choroid of enucleated globes of patients in chronic and convalescent stages [51].

Even when there is resolution of acute inflammation and recovery of good visual acuity, some patients still have subclinical retinal dysfunction as measured by multifocal electroretinogram (mfERG) [117, 118].

Some factors have been described as indicative of prognosis:

I. Related to treatment: a.. *Late* instauration *of treatment from the acute disease onset*: early treatment with high-dose systemic corticosteroids resulted in less persistent inflammation [176]; b.. *Treatment shorter than 6 months*: use of systemic corticosteroids for longer than 6 months and slow tapering were significantly associated with good final visual acuity [10, 127, 176]; c. *Treatment with corticosteroids in suboptimal dose*: patients treated with low doses of corticosteroids in the acute stage were more likely to have persistent inflammation [120, 121]. The extent of pigmentary changes in patients seemed to be dependent on the amount of corticosteroid received during the acute stage of the disease. Administering an initial high-dose of corticosteroid may preserve more melanocytes and may reduce the extent of pigment damage [178].

II. Related to the patient: a. *Younger age* (*controversial*): age at onset of the disease has been differently linked to final VA. Poor prognosis has been associated with older age at onset of VKHD by some authors [121, 162] and with younger age at onset by others [142, 177]; b.. Presence of HLA-DRB1*0405/0410 is more common in patients with prolonged disease: Islam et al. investigated HLA-DR4 gene variations in 46 Japanese patients, 28 with the prolonged type and 18 with the nonprolonged type of VKHD. Significant differences were found in the DR4 gene variation in the two clinical subtypes. All the patients with the prolonged type had either the DRB1*0405 or DRB1*0410 variant, whereas 39 % of the patients with the nonprolonged type had neither of them. This difference in frequency was statistically highly significant. The authors concluded that DR4 gene variants differed significantly between the two subtypes of VKHD, suggesting that the clinical course of VKHD is determined partly by the patient’s HLA-DR gene variation [179].

III. Related to the disease: a.*. Poor visual acuity at presentation*: better VA at presentation is associated with a better final VA [162]. Chee et al. proposed that good VA at one month was associated with greater likelihood of good VA at 3 years [121]. Final VA of 20/200 or worse may be explained by the presence of extensive pigmentary changes and disruption in the fundus secondary to previous inflammation and serous retinal detachment without any other associated complications [162]. Some VKHD patients may still have concomitant visual field loss and subclinical retinal dysfunction caused by chorioretinal atrophy and pigmentary changes, despite having a final VA of 20/20 [117, 121, 162, 180]. Besides, peripapillary atrophy is associated with visual dysfunction compared with eyes without peripapillary atrophy [117]. Early pinpoint peripapillary hyperfluorescence on pretreatment FA was found to be an indicator for good prognosis. In fact, this sign was more likely to be associated with eyes imaged early in the course of the disease than eyes imaged later [181]; b.*. Presence of complications in the initial presentation*: the development of ocular complications is significantly associated with a worse final VA [177]; c.. *Increased number of recurrences*: a longer duration of disease and higher number of recurrent episodes of inflammation are associated with a higher risk of complications and worse visual prognosis. A longer duration of disease and increased number of recurrences expose the eye to the harmful effects of active inflammation as well as treatment, especially corticosteroids [162].

## Conclusions

VKHD is a severe bilateral, granulomatous panuveitis associated with serous retinal detachment, causing a significant impact for patient’s life, especially considering its frequent onset at young and working ages. Ocular involvement undoubtedly accounts for most of the disease impact on individuals’ lives. While meningeal (neck stiffness, headache and CSF pleocytosis) and ocular signs are characteristic of acute stage, skin changes may be observed later on in the course of disease. Early diagnosis coupled with proper treatment may result in visual recovery. However, VKHD does require regular and close monitoring even in apparently quiescent cases as recent evidences lead to progressive subclinical visual deterioration in such instances.

### Ethical approval

All studies included in this review had the Ethics in Research Committee (CAPPesq) approval (#0496/2011).

## Consent

Written informed consent was obtained from the patient for the publication of this report and any accompanying images.

## Abbreviations

APMPPE: acute posterior multifocal placoid pigment epitheliopathy
AUS: American Uveitis Society
BL-FAF: blue fundus autofluorescence imaging
CME: cystoid macular edema
CNS: central nervous system
CNSNHF: CNS non-Hodgkin lymphoma
CNV: choroidal neovascularization
CSF: cerebrospinal fluid
EDI-OCT: enhanced depth imaging optical coherence tomography
ERG: electroretinography
FA: fluorescein angiography
FAF: fundus autofluorescence imaging
ffERG: full field electroretinography
HLA: human leukocyte antigen
ICGA: indocyanine green angiography
IMT: immunosuppressive therapy
IOL: intraocular lens
IOP: intraocular pressure
IRBP: interphotoreceptor retinoid binding protein
MCP: multifocal choroiditis and panuveitis
MEWDS: multiple evanescent white dot syndrome
mfERG: multifocal electroretinography
MHC: major histocompatibility complex
MRI: magnetic resonance imaging
NIR-FAF: near-infrared light fundus autofluorescence imaging
OCT: optical coherence tomography
PBMC: peripheral blood mononuclear cells
PCR: polymerase chain reaction
PPA: peripapillary atrophy
PSIIS: posterior segment imaging inflammatory signs
PVD: posterior vitreous detachment
RDC: revised diagnostic criteria
RPE: retinal pigment epithelium
RR: relative risk
TRP1: tyrosinase-related protein 1
TYR: tyrosinase
UBM: ultrasound biomicroscopy
US: ultrasonography
VA: visual acuity
VEGF: vascular endothelial growth factor
VKHD: Vogt-Koyanagi-Harada disease

## References

[1] Moorthy RS, Inomata H, Rao NA (1995). Vogt-Koyanagi-Harada syndrome. Surv Ophthalmol.

[2] Herbort CP, Mochizuki M (2007). Vogt-Koyanagi-Harada disease: inquiry into the genesis of a disease name in the historical context of Switzerland and Japan. Int Ophthalmol.

[3] Marmor MF, Ravin JG (2009). Chapter 34 - Goya’s Strange Malady. The Artist’s Eyes - Vision and the History of Art.

[4] Rao NA, Gupta A, Dustin L, Chee SP, Okada AA, Khairallah M (2010). Frequency of distinguishing clinical features in Vogt-Koyanagi-Harada disease. Ophthalmology.

[5] Ohguro N, Sonoda KH, Takeuchi M, Matsumura M, Mochizuki M (2012). The 2009 prospective multi-center epidemiologic survey of uveitis in Japan. Jpn J Ophthalmol.

[6] Ohno S, Char DH, Kimura SJ, O’Connor GR (1977). Vogt-Koyanagi-Harada syndrome. Am J Ophthalmol.

[7] Gouveia EB, Yamamoto JH, Abdalla M, Hirata CE, Kubo P, Olivalves E (2004). Causas das uveítes em serviço terciário em São Paulo, Brasil. Arq Bras Oftalmol.

[8] Belfort R, Nishi M, Hayashi S, Abreu MT, Petrilli AM, Plut RC (1988). Vogt-Koyanagi-Harada’s disease in Brazil. Jpn J Ophthalmol.

[9] Yang P, Zhang Z, Zhou H, Li B, Huang X, Gao Y (2005). Clinical patterns and characteristics of uveitis in a tertiary center for uveitis in China. Curr Eye Res.

[10] Rubsamen PE, Gass JD (1991). Vogt-Koyanagi-Harada syndrome. Clinical course, therapy, and long-term visual outcome. Arch Ophthalmol.

[11] Martin TD, Rathinam SR, Cunningham ET (2010). Prevalence, clinical characteristics, and causes of vision loss in children with Vogt-Koyanagi-Harada disease in South India. Retina.

[12] Hamade IH, Al Shamsi HN, Al Dhibi H, Chacra CB, Abu El-Asrar AM, Tabbara KF (2009). Uveitis survey in children. Br J Ophthalmol.

[13] Ikeda N, Hayasaka S, Hayasaka Y (2005). Uveitis and pseudouveitis presenting for the first time in Japanese elderly patients. Ophthalmologica.

[14] Kiyomoto C, Imaizumi M, Kimoto K, Abe H, Nakano S, Nakatsuka K (2007). Vogt-Koyanagi-Harada disease in elderly Japanese patients. Int Ophthalmol.

[15] Bassili SS, Peyman GA, Gebhardt BM, Daun M, Ganiban GJ, Rifai A (1996). Detection of Epstein-Barr virus DNA by polymerase chain reaction in the vitreous from a patient with Vogt-Koyanagi-Harada syndrome. Retina.

[16] Sugita S, Takase H, Kawaguchi T, Taguchi C, Mochizuki M (2007). Cross-reaction between tyrosinase peptides and cytomegalovirus antigen by T cells from patients with Vogt-Koyanagi-Harada disease. Int Ophthalmol.

[17] Matsuda H (1970). Electron microscopic studies on Vogt-Koyanagi-Harada syndrome and sympathetic ophthalmia with special reference to the melanocyte. Nihon Ganka Gakkai Zasshi.

[18] Hammer H (1974). Cellular hypersensitivity to uveal pigment confirmed by leucocyte migration tests in sympathetic ophthalmitis and the Vogt-Koyanagi-Harada syndrome. Br J Ophthalmol.

[19] Maezawa N, Yano A (1984). Two distinct cytotoxic T lymphocyte subpopulations in patients with Vogt-Koyanagi-Harada disease that recognize human melanoma cells. Microbiol Immunol.

[20] Norose K, Yano A, Aosai F, Segawa K (1990). Immunologic analysis of cerebrospinal fluid lymphocytes in Vogt-Koyanagi-Harada disease. Invest Ophthalmol Vis Sci.

[21] McClellan KA, MacDonald M, Hersey P, Billson FA (1989). Vogt-Koyanagi-Harada syndrome--isolation of cloned T cells with specificity for melanocytes and melanoma cells. Aust N Z J Ophthalmol.

[22] Kawakami Y, Robbins PF, Rosenberg SA (1996). Human melanoma antigens recognized by T lymphocytes. Keio J Med.

[23] Yamaki K, Gocho K, Hayakawa K, Kondo I, Sakuragi S (2000). Tyrosinase family proteins are antigens specific to Vogt-Koyanagi-Harada disease. J Immunol.

[24] Gocho K, Kondo I, Yamaki K (2001). Identification of autoreactive T cells in Vogt-Koyanagi-Harada disease. Invest Ophthalmol Vis Sci.

[25] Sugita S, Takase H, Taguchi C, Imai Y, Kamoi K, Kawaguchi T (2006). Ocular infiltrating CD4+ T cells from patients with Vogt-Koyanagi-Harada disease recognize human melanocyte antigens. Invest Ophthalmol Vis Sci.

[26] Damico FM, Cunha-Neto E, Goldberg AC, Iwai LK, Marin ML, Hammer J (2005). T-cell recognition and cytokine profile induced by melanocyte epitopes in patients with HLA-DRB1*0405-positive and -negative Vogt-Koyanagi-Harada uveitis. Invest Ophthalmol Vis Sci.

[27] Yamaki K, Kondo I, Nakamura H, Miyano M, Konno S, Sakuragi S (2000). Ocular and extraocular inflammation induced by immunization of tyrosinase related protein 1 and 2 in Lewis rats. Exp Eye Res.

[28] Sakamoto T, Murata T, Inomata H (1991). Class II major histocompatibility complex on melanocytes of Vogt-Koyanagi-Harada disease. Arch Ophthalmol.

[29] Inomata H, Sakamoto T (1990). Immunohistochemical studies of Vogt-Koyanagi-Harada disease with sunset sky fundus. Curr Eye Res.

[30] Davis JL, Mittal KK, Freidlin V, Mellow SR, Optican DC, Palestine AG (1990). HLA associations and ancestry in Vogt-Koyanagi-Harada disease and sympathetic ophthalmia. Ophthalmology.

[31] Tagawa Y, Sugiura S, Yakura H, Wakisaka A, Aizawa M (1976). The association between major histocompatibility antigens (HLA) and Vogt-Koyanagi-Harada syndrome (author’s transl). Nihon Ganka Gakkai Zasshi.

[32] Shindo Y, Inoko H, Yamamoto T, Ohno S (1994). HLA-DRB1 typing of Vogt-Koyanagi-Harada’s disease by PCR-RFLP and the strong association with DRB1*0405 and DRB1*0410. Br J Ophthalmol.

[33] Islam SM, Numaga J, Fujino Y, Hirata R, Matsuki K, Maeda H (1994). HLA class II genes in Vogt-Koyanagi-Harada disease. Invest Ophthalmol Vis Sci.

[34] Zhang XY, Wang XM, Hu TS (1992). Profiling human leukocyte antigens in Vogt-Koyanagi-Harada syndrome. Am J Ophthalmol.

[35] Weisz JM, Holland GN, Roer LN, Park MS, Yuge AJ, Moorthy RS (1995). Association between Vogt-Koyanagi-Harada syndrome and HLA-DR1 and -DR4 in Hispanic patients living in southern California. Ophthalmology.

[36] Goldberg AC, Yamamoto JH, Chiarella JM, Marin ML, Sibinelli M, Neufeld R (1998). HLA-DRB1*0405 is the predominant allele in Brazilian patients with Vogt-Koyanagi-Harada disease. Hum Immunol.

[37] Ng JY, Luk FO, Lai TY, Pang CP (2014). Influence of molecular genetics in Vogt-Koyanagi-Harada disease. J Ophthalmic Inflamm Infect.

[38] Hou S, Du L, Lei B, Pang CP, Zhang M, Zhuang W (2014). Genome-wide association analysis of Vogt-Koyanagi-Harada syndrome identifies two new susceptibility loci at 1p31.2 and 10q21.3. Nat Genet.

[39] Rao NA (1997). Mechanisms of inflammatory response in sympathetic ophthalmia and VKH syndrome. Eye (Lond).

[40] Damico FM, Bezerra FT, Silva GC, Gasparin F, Yamamoto JH (2009). New insights into Vogt-Koyanagi-Harada disease. Arq Bras Oftalmol.

[41] Imai Y, Sugita M, Nakamura S, Toriyama S, Ohno S (2001). Cytokine production and helper T cell subsets in Vogt-Koyanagi-Harada’s disease. Curr Eye Res.

[42] Chi W, Yang P, Li B, Wu C, Jin H, Zhu X (2007). IL-23 promotes CD4+ T cells to produce IL-17 in Vogt-Koyanagi-Harada disease. J Allergy Clin Immunol.

[43] Shu Q, Yang P, Hou S, Li F, Chen Y, Du L (2010). Interleukin-17 gene polymorphism is associated with Vogt-Koyanagi-Harada syndrome but not with Behcet’s disease in a Chinese Han population. Hum Immunol.

[44] Li F, Yang P, Liu X, Wang C, Hou S, Kijlstra A (2010). Upregulation of interleukin 21 and promotion of interleukin 17 production in chronic or recurrent Vogt-Koyanagi-Harada disease. Arch Ophthalmol.

[45] Wang C, Tian Y, Lei B, Xiao X, Ye Z, Li F (2012). Decreased IL-27 expression in association with an increased Th17 response in Vogt-Koyanagi-Harada disease. Invest Ophthalmol Vis Sci.

[46] Commodaro AG, Peron JP, Genre J, Arslanian C, Sanches L, Muccioli C (2010). IL-10 and TGF-beta immunoregulatory cytokines rather than natural regulatory T cells are associated with the resolution phase of Vogt-Koyanagi-Harada (VKH) syndrome. Scand J Immunol.

[47] Chen L, Yang P, Zhou H, He H, Ren X, Chi W (2008). Diminished frequency and function of CD4 + CD25high regulatory T cells associated with active uveitis in Vogt-Koyanagi-Harada syndrome. Invest Ophthalmol Vis Sci.

[48] Chen W, Lin H, Zhong X, Liu Z, Geng Y, Xie C (2014). Discrepant expression of cytokines in inflammation- and age-related cataract patients. PLoS One.

[49] Naidu YM, Pararajasegaram G, Sun Y (1991). Predominant expression of T-cell antigen receptor (TCR) Vα 10 in Vogt-Koyanagi-Harada (VKH) syndrome. Invest Ophthalmol Vis Sci.

[50] de Smet MD, Yamamoto JH, Mochizuki M, Gery I, Singh VK, Shinohara T (1990). Cellular immune responses of patients with uveitis to retinal antigens and their fragments. Am J Ophthalmol.

[51] Chan CC, Palestine AG, Nussenblatt RB, Roberge FG, Benezra D (1985). Anti-retinal auto-antibodies in Vogt-Koyanagi-Harada syndrome, Behcet’s disease, and sympathetic ophthalmia. Ophthalmology.

[52] Shinzato M, Yamamoto JH, Hirata CE, Olivalves E, Bonfa E (2004). Anti-SS-A/Ro reactivity in patients with Vogt-Koyanagi-Harada syndrome. Lupus.

[53] Rao NA (2007). Pathology of Vogt-Koyanagi-Harada disease. Int Ophthalmol.

[54] Inomata H, Rao NA (2001). Depigmented atrophic lesions in sunset glow fundi of Vogt-Koyanagi-Harada disease. Am J Ophthalmol.

[55] Morohashi M, Hashimoto K, Goodman TF, Newton DE, Rist T (1977). Ultrastructural studies of vitiligo, Vogt-Koyanagi syndrome, and incontinentia pigmenti achromians. Arch Dermatol.

[56] Snyder DA, Tessler HH (1980). Vogt-Koyanagi-Harada syndrome. Am J Ophthalmol.

[57] Sugiura S (1976). Some observations on uveitis in Japan, with special reference to Vogt-Koyanagi-Harada and Behcet diseases (author’s transl). Nihon Ganka Gakkai Zasshi.

[58] Sugiura S (1978). Vogt-Koyanagi-Harada Disease. Jpn J Ophthalmol.

[59] Read RW, Holland GN, Rao NA, Tabbara KF, Ohno S, Arellanes-Garcia L (2001). Revised diagnostic criteria for Vogt-Koyanagi-Harada disease: report of an international committee on nomenclature. Am J Ophthalmol.

[60] Herbort CP, LeHoang P, Guex-Crosier Y (1998). Schematic interpretation of indocyanine green angiography in posterior uveitis using a standard angiographic protocol. Ophthalmology.

[61] Herbort CP, Mantovani A, Bouchenaki N (2007). Indocyanine green angiography in Vogt-Koyanagi-Harada disease: angiographic signs and utility in patient follow-up. Int Ophthalmol.

[62] Fardeau C, Tran TH, Gharbi B, Cassoux N, Bodaghi B, LeHoang P (2007). Retinal fluorescein and indocyanine green angiography and optical coherence tomography in successive stages of Vogt-Koyanagi-Harada disease. Int Ophthalmol.

[63] da Silva FT, Damico FM, Marin ML, Goldberg AC, Hirata CE, Takiuti PH (2009). Revised diagnostic criteria for vogt-koyanagi-harada disease: considerations on the different disease categories. Am J Ophthalmol.

[64] da Silva FT, Hirata CE, Olivalves E, Oyamada MK, Yamamoto JH (2009). Fundus-based and electroretinographic strategies for stratification of late-stage Vogt-Koyanagi-Harada disease patients. Am J Ophthalmol.

[65] Al Dousary S (2011). Auditory and vestibular manifestations of Vogt-Koyanagi-Harada disease. J Laryngol Otol.

[66] Yang P, Ren Y, Li B, Fang W, Meng Q, Kijlstra A (2007). Clinical characteristics of Vogt-Koyanagi-Harada syndrome in Chinese patients. Ophthalmology.

[67] Usui Y, Goto H, Sakai J, Takeuchi M, Usui M, Rao NA (2009). Presumed Vogt-Koyanagi-Harada disease with unilateral ocular involvement: report of three cases. Graefes Arch Clin Exp Ophthalmol.

[68] Sakata VM, da Silva FT, Hirata CE, Marin ML, Rodrigues H, Kalil J (2015). High rate of clinical recurrence in patients with Vogt-Koyanagi-Harada disease treated with early high-dose corticosteroids. Graefes Arch Clin Exp Ophthalmol.

[69] Knecht PB, Mantovani A, Herbort CP (2013). Indocyanine green angiography-guided management of Vogt-Koyanagi-Harada disease: differentiation between choroidal scars and active lesions. Int Ophthalmol.

[70] da Silva FT, Hirata CE, Sakata VM, Olivalves E, Preti R, Pimentel SL (2012). Indocyanine green angiography findings in patients with long-standing Vogt-Koyanagi-Harada disease: a cross-sectional study. BMC Ophthalmol.

[71] Gu S, Liu Y, Song Z, Zi X, Deng H (2013). Acute myelitis in a patient with vogt-koyanagi-harada disease: case report and review of the literature. J Clin Neurol.

[72] Beniz J, Forster DJ, Lean JS, Smith RE, Rao NA (1991). Variations in clinical features of the Vogt-Koyanagi-Harada syndrome. Retina.

[73] Barnes L (1988). Vitiligo and the Vogt-Koyanagi-Harada syndrome. Dermatol Clin.

[74] Friedman AH, Deutsch-Sokol RH (1981). Sugiura’s sign. Perilimbal vitiligo in the Vogt-Koyanagi-Harada syndrome. Ophthalmology.

[75] Fiore T, Iaccheri B, Androudi S, Papadaki TG, Anzaar F, Brazitikos P (2009). Acute posterior multifocal placoid pigment epitheliopathy: outcome and visual prognosis. Retina.

[76] Gill MK, Jampol LM (2001). Variations in the presentation of primary intraocular lymphoma: case reports and a review. Surv Ophthalmol.

[77] Gross NE, Yannuzzi LA, Freund KB, Spaide RF, Amato GP, Sigal R (2006). Multiple evanescent white dot syndrome. Arch Ophthalmol.

[78] Levinson RD, Brezin A, Rothova A, Accorinti M, Holland GN (2006). Research criteria for the diagnosis of birdshot chorioretinopathy: results of an international consensus conference. Am J Ophthalmol.

[79] McCluskey PJ, Watson PG, Lightman S, Haybittle J, Restori M, Branley M (1999). Posterior scleritis: clinical features, systemic associations, and outcome in a large series of patients. Ophthalmology.

[80] Vianna RN, Ozdal PC, Filho JP, Ventura MP, Saraiva VS, Deschenes J (2004). Longterm follow-up of patients with multifocal choroiditis and panuveitis. Acta Ophthalmol Scand.

[81] Nguyen QD, Uy HS, Akpek EK, Harper SL, Zacks DN, Foster CS (2000). Choroidopathy of systemic lupus erythematosus. Lupus.

[82] Elagouz M, Stanescu-Segall D, Jackson TL (2010). Uveal effusion syndrome. Surv Ophthalmol.

[83] Rao NA, Marak GE (1983). Sympathetic ophthalmia simulating vogt-Koyanagi-Harada’s disease: a clinico-pathologic study of four cases. Jpn J Ophthalmol.

[84] Gass JD (1968). Acute posterior multifocal placoid pigment epitheliopathy. Arch Ophthalmol.

[85] Steiner S, Goldstein DA (2012). Imaging in the diagnosis and management of APMPPE. Int Ophthalmol Clin.

[86] Lee GE, Lee BW, Rao NA, Fawzi AA (2011). Spectral domain optical coherence tomography and autofluorescence in a case of acute posterior multifocal placoid pigment epitheliopathy mimicking Vogt-Koyanagi-Harada disease: case report and review of literature. Ocul Immunol Inflamm.

[87] Aoyagi R, Hayashi T, Masai A, Mitooka K, Gekka T, Kozaki K (2012). Subfoveal choroidal thickness in multiple evanescent white dot syndrome. Clin. Exp. Optom..

[88] Vance SK, Khan S, Klancnik JM, Freund KB (2011). Characteristic spectral-domain optical coherence tomography findings of multifocal choroiditis. Retina.

[89] Benson WE (1988). Posterior scleritis. Surv Ophthalmol.

[90] Carvalho-Recchia CA, Yannuzzi LA, Negrao S, Spaide RF, Freund KB, Rodriguez-Coleman H (2002). Corticosteroids and central serous chorioretinopathy. Ophthalmology.

[91] Shin WB, Kim MK, Lee CS, Lee SC, Kim H (2015). Comparison of the clinical manifestations between acute Vogt-Koyanagi-Harada disease and acute bilateral central serous chorioretinopathy. Korean J Ophthalmol.

[92] Wolfensberger TJ, Tufail A (2000). Systemic disorders associated with detachment of the neurosensory retina and retinal pigment epithelium. Curr Opin Ophthalmol.

[93] Aisenbrey S, Luke C, Ayertey HD, Grisanti S, Perniok A, Brunner R (2003). Vogt-Koyanagi-Harada syndrome associated with cutaneous malignant melanoma: an 11-year follow-up. Graefes Arch Clin Exp Ophthalmol.

[94] Souguir A, Hammami A, Dahmeni W, Jaziri H, Ben Mansour I, Zayene A, et al. Vogt-Koyanagi-Harada syndrome and Crohn’s disease: an exceptional association. Gastroenterology report. 2015. [Epub ahead of print] 10.1093/gastro/gov056 PMC5806415 26534928

[95] Federman DG, Kravetz JD, Ruser CB, Judson PH, Kirsner RS (2004). Vogt-Koyanagi-Harada syndrome and ulcerative colitis. South Med J.

[96] Kaya MK, Turgut B, Demir T, Celiker U, Gurates B (2011). A case of vogt-koyanagi-harada disease associated with polycystic ovary syndrome. J Clin Med Res.

[97] Sakata VM, da Silva FT, Hirata CE, de Carvalho JF, Yamamoto JH (2014). Diagnosis and classification of Vogt-Koyanagi-Harada disease. Autoimmun Rev.

[98] Arellanes-Garcia L, Hernandez-Barrios M, Fromow-Guerra J, Cervantes-Fanning P (2007). Fluorescein fundus angiographic findings in Vogt-Koyanagi-Harada syndrome. Int Ophthalmol.

[99] Stanga PE, Lim JI, Hamilton P (2003). Indocyanine green angiography in chorioretinal diseases: indications and interpretation: an evidence-based update. Ophthalmology.

[100] Koizumi H, Maruyama K, Kinoshita S (2010). Blue light and near-infrared fundus autofluorescence in acute Vogt-Koyanagi-Harada disease. Br J Ophthalmol.

[101] Heussen FM, Vasconcelos-Santos DV, Pappuru RR, Walsh AC, Rao NA, Sadda SR (2011). Ultra-wide-field green-light (532-nm) autofluorescence imaging in chronic Vogt-Koyanagi-Harada disease. Ophthalmic Surg Lasers Imaging.

[102] Ishihara K, Hangai M, Kita M, Yoshimura N (2009). Acute Vogt-Koyanagi-Harada disease in enhanced spectral-domain optical coherence tomography. Ophthalmology.

[103] Nazari H, Rao NA (2012). Resolution of subretinal fluid with systemic corticosteroid treatment in acute Vogt-Koyanagi-Harada disease. Br J Ophthalmol.

[104] Zhao C, Zhang MF, Dong FT, Wang XQ, Wen X, Dai RP (2012). Spectral domain optical coherence tomography of Vogt-Koyanagi-Harada disease: novel findings and new insights into the pathogenesis. Chin. Med. Sci. J.

[105] Ikewaki J, Kimoto K, Choshi T, Nagata M, Motomura Y, Tamura K (2011). Optical coherence tomographic assessment of dynamic macular changes in patients with Vogt-Koyanagi-Harada disease. Int Ophthalmol.

[106] Gupta V, Gupta A, Gupta P, Sharma A (2009). Spectral-domain cirrus optical coherence tomography of choroidal striations seen in the acute stage of Vogt-Koyanagi-Harada disease. Am J Ophthalmol.

[107] Fong AH, Li KK, Wong D (2011). Choroidal evaluation using enhanced depth imaging spectral-domain optical coherence tomography in Vogt-Koyanagi-Harada disease. Retina.

[108] Yamanaka E, Ohguro N, Yamamoto S, Nakagawa Y, Imoto Y, Tano Y (2002). Evaluation of pulse corticosteroid therapy for vogt-koyanagi-harada disease assessed by optical coherence tomography. Am J Ophthalmol.

[109] Vasconcelos-Santos DV, Sohn EH, Sadda S, Rao NA (2010). Retinal pigment epithelial changes in chronic Vogt-Koyanagi-Harada disease: fundus autofluorescence and spectral domain-optical coherence tomography findings. Retina.

[110] Margolis R, Spaide RF (2009). A pilot study of enhanced depth imaging optical coherence tomography of the choroid in normal eyes. Am J Ophthalmol.

[111] Nakayama M, Keino H, Okada AA, Watanabe T, Taki W, Inoue M (2012). Enhanced depth imaging optical coherence tomography of the choroid in Vogt-Koyanagi-Harada disease. Retina.

[112] Maruko I, Iida T, Sugano Y, Oyamada H, Sekiryu T, Fujiwara T (2011). Subfoveal choroidal thickness after treatment of Vogt-Koyanagi-Harada disease. Retina.

[113] da Silva FT, Sakata VM, Nakashima A, Hirata CE, Olivalves E, Takahashi WY (2013). Enhanced depth imaging optical coherence tomography in long-standing Vogt-Koyanagi-Harada disease. Br J Ophthalmol.

[114] Hashizume K, Imamura Y, Fujiwara T, Machida S, Ishida M, Kurosaka D (2014). Choroidal thickness in eyes with posterior recurrence of Vogt-Koyanagi-Harada disease after high-dose steroid therapy. Acta Ophthalmol.

[115] Forster DJ, Cano MR, Green RL, Rao NA (1990). Echographic features of the Vogt-Koyanagi-Harada syndrome. Arch Ophthalmol.

[116] Gohdo T, Tsukahara S (1996). Ultrasound Biomicroscopy of shallow anterior chamber in Vogt-Koyanagi-Harada syndrome. Am J Ophthalmol.

[117] Chee SP, Luu CD, Cheng CL, Lim WK, Jap A (2005). Visual function in Vogt-Koyanagi-Harada patients. Graefes Arch Clin Exp Ophthalmol.

[118] Yang P, Fang W, Wang L, Wen F, Wu W, Kijlstra A (2008). Study of macular function by multifocal electroretinography in patients with Vogt-Koyanagi-Harada syndrome. Am J Ophthalmol.

[119] Read RW, Yu F, Accorinti M, Bodaghi B, Chee SP, Fardeau C (2006). Evaluation of the effect on outcomes of the route of administration of corticosteroids in acute Vogt-Koyanagi-Harada disease. Am J Ophthalmol.

[120] Kawaguchi T, Horie S, Bouchenaki N, Ohno-Matsui K, Mochizuki M, Herbort CP (2010). Suboptimal therapy controls clinically apparent disease but not subclinical progression of Vogt-Koyanagi-Harada disease. Int Ophthalmol.

[121] Chee SP, Jap A, Bacsal K (2009). Prognostic factors of Vogt-Koyanagi-Harada disease in Singapore. Am J Ophthalmol.

[122] Rao NA (2006). Treatment of Vogt-Koyanagi-Harada disease by corticosteroids and immunosuppressive agents. Ocul Immunol Inflamm.

[123] Chee S-P, Jap A (2013). The outcomes of indocyanine green angiography monitored immunotherapy in Vogt-Koyanagi-Harada disease. Br J Ophthalmol.

[124] Lai TY, Chan RP, Chan CK, Lam DS (2009). Effects of the duration of initial oral corticosteroid treatment on the recurrence of inflammation in Vogt-Koyanagi-Harada disease. Eye (Lond).

[125] Errera MH, Fardeau C, Cohen D, Navarro A, Gaudric A, Bodaghi B (2011). Effect of the duration of immunomodulatory therapy on the clinical features of recurrent episodes in Vogt--Koyanagi--Harada disease. Acta Ophthalmol.

[126] Jabs DA, Rosenbaum JT, Foster CS, Holland GN, Jaffe GJ, Louie JS (2000). Guidelines for the use of immunosuppressive drugs in patients with ocular inflammatory disorders: recommendations of an expert panel. Am J Ophthalmol.

[127] Kempen JH, Gangaputra S, Daniel E, Levy-Clarke GA, Nussenblatt RB, Rosenbaum JT (2008). Long-term risk of malignancy among patients treated with immunosuppressive agents for ocular inflammation: a critical assessment of the evidence. Am J Ophthalmol.

[128] Paredes I, Ahmed M, Foster CS (2006). Immunomodulatory therapy for Vogt-Koyanagi-Harada patients as first-line therapy. Ocul Immunol Inflamm.

[129] Kacmaz RO, Kempen JH, Newcomb C, Daniel E, Gangaputra S, Nussenblatt RB (2010). Cyclosporine for ocular inflammatory diseases. Ophthalmology.

[130] Galor A, Jabs DA, Leder HA, Kedhar SR, Dunn JP, Peters GB (2008). Comparison of antimetabolite drugs as corticosteroid-sparing therapy for noninfectious ocular inflammation. Ophthalmology.

[131] Kondo Y, Fukuda K, Suzuki K, Nishida T (2012). Chronic noninfectious uveitis associated with Vogt-Koyanagi-Harada disease treated with low-dose weekly systemic methotrexate. Jpn J Ophthalmol.

[132] Abu El-Asrar AM, Hemachandran S, Al-Mezaine HS, Kangave D, Al-Muammar AM (2012). The outcomes of mycophenolate mofetil therapy combined with systemic corticosteroids in acute uveitis associated with Vogt-Koyanagi-Harada disease. Acta Ophthalmol.

[133] Urzua CA, Velasquez V, Sabat P, Berger O, Ramirez S, Goecke A (2015). Earlier immunomodulatory treatment is associated with better visual outcomes in a subset of patients with Vogt-Koyanagi-Harada disease. Acta Ophthalmol.

[134] Kim SJ, Yu HG (2007). The use of low-dose azathioprine in patients with Vogt-Koyanagi-Harada disease. Ocul Immunol Inflamm.

[135] Wang Y, Gaudio PA (2008). Infliximab therapy for 2 patients with Vogt-Koyanagi-Harada syndrome. Ocul Immunol Inflamm.

[136] Niccoli L, Nannini C, Cassara E, Gini G, Lenzetti I, Cantini F (2009). Efficacy of infliximab therapy in two patients with refractory Vogt-Koyanagi-Harada disease. Br J Ophthalmol.

[137] Dolz-Marco R, Gallego-Pinazo R, Diaz-Llopis M (2011). Rituximab in refractory Vogt-Koyanagi-Harada disease. J Ophthalmic Inflamm Infect.

[138] Andrade RE, Muccioli C, Farah ME, Nussenblatt RB, Belfort R (2004). Intravitreal triamcinolone in the treatment of serous retinal detachment in Vogt-Koyanagi-Harada syndrome. Am J Ophthalmol.

[139] Karacorlu M, Arf Karacorlu S, Ozdemir H (2006). Intravitreal triamcinolone acetonide in Vogt-Koyanagi-Harada syndrome. Eur J Ophthalmol.

[140] Park HS, Nam KY, Kim JY (2011). Intravitreal bevacizumab injection for persistent serous retinal detachment associated with Vogt-Koyanagi-Harada disease. Graefes Arch Clin Exp Ophthalmol.

[141] Khalifa Y, Loh AR, Acharya NR (2009). Fluocinolone acetonide intravitreal implants in Vogt-Koyanagi-Harada disease. Ocul Immunol Inflamm.

[142] Tabbara KF, Chavis PS, Freeman WR (1998). Vogt-Koyanagi-Harada syndrome in children compared to adults. Acta Ophthalmol Scand.

[143] Soheilian M, Aletaha M, Yazdani S, Dehghan MH, Peyman GA (2006). Management of pediatric Vogt-Koyanagi- Harada (VKH)-associated panuveitis. Ocul Immunol Inflamm.

[144] Abu El-Asrar AM, Al-Kharashi AS, Aldibhi H, Al-Fraykh H, Kangave D (2008). Vogt-Koyanagi-Harada disease in children. Eye (Lond).

[145] Friedman Z, Granat M, Neumann E (1980). The syndrome of Vogt-Koyanagi-Harada and pregnancy. Metab Pediatr Ophthalmol.

[146] Miyata N, Sugita M, Nakamura S, Isobe K, Matoba H, Tsuda K (2001). Treatment of Vogt-Koyanagi- Harada’s disease during pregnancy. Jpn J Ophthalmol.

[147] Doi M, Matsubara H, Uji Y (2000). Vogt-Koyanagi-Harada syndrome in a pregnant patient treated with high-dose systemic corticosteroids. Acta Ophthalmol Scand.

[148] Tien MC, Teoh SC (2009). Treatment of Vogt-Koyanagi-Harada syndrome in pregnancy. Can J Ophthalmol.

[149] Onishi SM, Asahi MG, Chou C, Gallemore RP (2015). Topical difluprednate for the treatment of Harada’s disease. Clin Ophthalmol.

[150] Maya JR, Sadiq MA, Zapata LJ, Hanout M, Sarwar S, Rajagopalan N (2014). Emerging therapies for noninfectious uveitis: what may be coming to the clinics. J Ophthalmol.

[151] Rutzen AR, Ortega-Larrocea G, Frambach DA, Rao NA (1995). Macular edema in chronic Vogt-Koyanagi-Harada syndrome. Retina.

[152] Khairallah M, Rao NA, Ben Yahia S, Zaouali S, Attia S (2006). Pseudotumoral retinal pigment epithelium proliferation in a patient with Vogt-Koyanagi-Harada disease. Arch Ophthalmol.

[153] Yang P, Sun M (2011). Band-shaped keratopathy in Chinese patients with Vogt-Koyanagi-Harada syndrome. Cornea.

[154] Nakao K, Mizushima Y, Abematsu N, Goh N, Sakamoto T (2009). Anterior ischemic optic neuropathy associated with Vogt-Koyanagi-Harada disease. Graefes Arch Clin Exp Ophthalmol.

[155] Moorthy RS, Rajeev B, Smith RE, Rao NA (1994). Incidence and management of cataracts in Vogt-Koyanagi-Harada syndrome. Am J Ophthalmol.

[156] Mehta S, Linton MM, Kempen JH (2014). Outcomes of cataract surgery in patients with uveitis: a systematic review and meta-analysis. Am J Ophthalmol.

[157] Quek DT, Jap A, Chee SP (2011). Risk factors for poor visual outcome following cataract surgery in Vogt-Koyanagi-Harada disease. Br J Ophthalmol.

[158] Ganesh SK, Padmaja, Babu K, Biswas J (2004). Cataract surgery in patients with Vogt-Koyanagi-Harada syndrome. J Cataract Refract Surg.

[159] Meacock WR, Spalton DJ, Bender L, Antcliff R, Heatley C, Stanford MR (2004). Steroid prophylaxis in eyes with uveitis undergoing phacoemulsification. Br J Ophthalmol.

[160] Lundvall A, Zetterstrom C (2000). Cataract extraction and intraocular lens implantation in children with uveitis. Br J Ophthalmol.

[161] Ram J, Gupta A, Kumar S, Kaushik S, Gupta N, Severia S (2010). Phacoemulsification with intraocular lens implantation in patients with uveitis. J Cataract Refract Surg.

[162] Read RW, Rechodouni A, Butani N, Johnston R, LaBree LD, Smith RE (2001). Complications and prognostic factors in Vogt-Koyanagi-Harada disease. Am J Ophthalmol.

[163] Takahashi T, Ohtani S, Miyata K, Miyata N, Shirato S, Mochizuki M (2002). A clinical evaluation of uveitis-associated secondary glaucoma. Jpn J Ophthalmol.

[164] Iwao K, Inatani M, Seto T, Takihara Y, Ogata-Iwao M, Okinami S (2014). Long-term outcomes and prognostic factors for trabeculectomy with mitomycin C in eyes with uveitic glaucoma: a retrospective cohort study. J Glaucoma.

[165] Kolomeyer AM, Roy MS, Chu DS (2011). The use of intravitreal ranibizumab for choroidal neovascularization associated with vogt-koyanagi-harada syndrome. Case Rep Med.

[166] Wu L, Evans T, Saravia M, Schlaen A, Couto C (2009). Intravitreal bevacizumab for choroidal neovascularization secondary to Vogt-Koyanagi-Harada syndrome. Jpn J Ophthalmol.

[167] Mansour AM, Arevalo JF, Ziemssen F, Mehio-Sibai A, Mackensen F, Adan A (2009). Long-term visual outcomes of intravitreal bevacizumab in inflammatory ocular neovascularization. Am J Ophthalmol.

[168] Nowilaty SR, Bouhaimed M (2006). Photodynamic therapy for subfoveal choroidal neovascularisation in Vogt-Koyanagi-Harada disease. Br J Ophthalmol.

[169] Soheilian M, Movaseghi M, Ramezani A, Peyman GA (2011). Pilot study of safety and effect of combined intravitreal bevacizumab and methotrexate for neovascular age-related macular degeneration. Eur J Ophthalmol.

[170] Lertsumitkul S, Whitcup SM, Nussenblatt RB, Chan CC (1999). Subretinal fibrosis and choroidal neovascularization in Vogt-Koyanagi-Harada syndrome. Graefes Arch Clin Exp Ophthalmol.

[171] Lertsumitkul S, Whitcup SM, Chan CC, Nussenblatt RB (2001). Subretinal fibrosis in Vogt-Koyanagi Harada syndrome. Ophthalmology.

[172] Kuo IC, Rechdouni A, Rao NA, Johnston RH, Margolis TP, Cunningham ET (2000). Subretinal fibrosis in patients with Vogt-Koyanagi-Harada disease. Ophthalmology.

[173] Ozdal P, Ozdamar Y, Yazici A, Teke MY, Ozturk F (2014). Vogt-Koyanagi-Harada disease: clinical and demographic characteristics of patients in a specialized eye hospital in Turkey. Ocul Immunol Inflamm.

[174] Bacsal K, Wen DSH, Chee S-P (2008). Concomitant choroidal inflammation during anterior segment recurrence in Vogt-Koyanagi-Harada disease. Am J Ophthalmol.

[175] Keino H, Goto H, Usui M (2002). Sunset glow fundus in Vogt-Koyanagi-Harada disease with or without chronic ocular inflammation. Graefes Arch Clin Exp Ophthalmol.

[176] Ohno S, Minakawa R, Matsuda H (1988). Clinical studies of Vogt-Koyanagi-Harada’s disease. Jpn J Ophthalmol.

[177] Al-Kharashi AS, Aldibhi H, Al-Fraykh H, Kangave D, Abu El-Asrar AM (2007). Prognostic factors in Vogt-Koyanagi-Harada disease. Int Ophthalmol.

[178] Jap A, Luu CD, Yeo I, Chee SP (2008). Correlation between peripapillary atrophy and corticosteroid therapy in patients with Vogt-Koyanagi-Harada disease. Eye (Lond).

[179] Islam SM, Numaga J, Matsuki K, Fujino Y, Maeda H, Masuda K (1994). Influence of HLA-DRB1 gene variation on the clinical course of Vogt-Koyanagi-Harada disease. Invest Ophthalmol Vis Sci.

[180] Sonoda S, Nakao K, Ohba N (1999). Extensive chorioretinal atrophy in Vogt-Koyanagi-Harada disease. Jpn J Ophthalmol.

[181] Chee SP, Jap A, Cheung CM (2010). The prognostic value of angiography in Vogt-Koyanagi-Harada disease. Am J Ophthalmol.

[182] Horie Y, Kitaichi N, Takemoto Y, Namba K, Yoshida K, Hirose S (2007). Polymorphism of IFNgamma gene and Vogt-Koyanagi-Harada disease. Mol Vis.

[183] Forster DJ, Rao NA, Hill RA, Nguyen QH, Baerveldt G (1993). Incidence and management of glaucoma in Vogt-Koyanagi-Harada syndrome. Ophthalmology.

